# Genetic dissection of host immune response in pneumonia development and progression

**DOI:** 10.1038/srep35021

**Published:** 2016-10-11

**Authors:** Tamara V. Smelaya, Olesya B. Belopolskaya, Svetlana V. Smirnova, Artem N. Kuzovlev, Viktor V. Moroz, Arkadiy M. Golubev, Noel A. Pabalan, Lyubov E. Salnikova

**Affiliations:** 1 V. A. Negovsky Research Institute of General Reanimatology, Russian Academy of Sciences, 25 Petrovka str., build.2, Moscow 107031, Russia; 2 N.I. Vavilov Institute of General Genetics, Russian Academy of Sciences, 3 Gubkin street, Moscow 117971, Russia; 3 Center for Research and Development, Angeles University Foundation, Angeles City 2009, Philippines

## Abstract

The role of host genetic variation in pneumonia development and outcome is poorly understood. We studied common polymorphisms in the genes of proinflammatory cytokines (*IL6* rs1800795, *IL8* rs4073, *IL1B* rs16944), anti-inflammatory cytokines (*IL10* rs1800896, *IL4* rs2243250, *IL13* rs20541) and toll-like receptors (*TLR2* rs5743708 and rs4696480, *TLR4* rs4986791, *TLR9* rs352139, rs5743836 and rs187084) in patients with community-acquired pneumonia (CAP) (390 cases, 203 controls) and nosocomial pneumonia (355 cases, 216 controls). Experimental data were included in a series of 11 meta-analyses and eight subset analyses related to pneumonia susceptibility and outcome. *TLR2* rs5743708 minor genotype appeared to be associated with CAP/Legionnaires’ disease/pneumococcal disease. In CAP patients, the *IL6* rs1800795-C allele was associated with severe sepsis/septic shock/severe systemic inflammatory response, while the *IL10* rs1800896-A allele protected against the development of these critical conditions. To contribute to deciphering of the above results, we performed an *in silico* analysis and a qualitative synthesis of literature data addressing basal and stimulated genotype-specific expression level. This data together with database information on transcription factors’ affinity changes caused by SNPs in putative promoter regions, the results of linkage disequilibrium analysis along with SNPs functional annotations supported assumptions about the complexity underlying the revealed associations.

Pneumonia, one of the most common infectious diseases, is associated with high morbidity and mortality worldwide. Pneumonia is classified according to the site of acquisition as community-acquired (CAP) and hospital-acquired (or nosocomial) pneumonia (HAP or NP), which includes healthcare-associated pneumonia (HCAP) and ventilator-associated pneumonia (VAP). HAP tends to be a more serious problem than CAP since HAP patients have an increased risk of infection caused by antibiotic-resistant organisms, besides they are often older and burdened by comorbidities. Pneumonia is considered to be the the most prevalent cause of sepsis associated with the highest mortality rates^1^.

In infection, host genetic background may be of even more importance than in cancer and cardiovascular diseases which are known to have a high hereditary component^2^. Most infectious phenotypes result from a complex interplay between multiple genetic factors related to host and pathogen genetics and a variety of non-genetic factors. Since infectious diseases are related to immune-mediated disorders, genetic susceptibility loci for immune traits can be considered as good candidates in genetic association studies of pneumonia and its complications^3^. The first line of defence of the innate immune system is represented by pattern-recognition receptors that sense bacterial, viral or fungal molecular structures and induce systemic inflammation. The most studied class of pattern-recognition receptors is that of the toll-like receptors (TLRs). Once inflammation has been launched a crosstalk between pro-and anti-inflammatory branches of the immune response should provide a balanced immune reaction crucial for a favorable resolution of infection.

Genetic studies of pneumonia are not numerous while investigations assessing the role of host genetics in sepsis due to pneumonia are even more unfrequent. Furthermore, to date there are no GWAS reported for pneumonia. Meta-analyses of genetic susceptibility to pneumonia provided very limited coverage of common genetic variations being performed for the genes of *TNFa*^4^, *MBL2*^5^, *IL6*^6^,^7^, *IL10*^7^, and *FCGR2A*^6^ with no association effect and for the loci *ACE*^8^ and *TLR4*^9^ found to be associated with pneumonia. Several studies showed that genetic associations of sepsis in pneumonia differ from those in other infections^10^,^11^,^12^ and may in particular depend on a specific pathogen^13^.

Taking into account this scientific background the aim of our study was: (i) to investigate the role of the SNPs in the genes of proinflammatory cytokines (*IL6, IL1B, IL8*), antiinflammatory cytokines (*IL10, IL4, IL13*) and toll-like receptors *TLR2*, *TLR4* and *TLR9* in CAP and HAP patients, (ii) to combine existent knowledge and our own experimental data on the above genes in a series of meta-analyses of genetic susceptibility to pneumonia development and outcome. The relationship between host genetics and clinical phenotypes is mediated through a large number of factors and interactions, which are essentially a “black box”. To contribute to deciphering of this “black box” in terms of the studied genes several additional investigations were performed. We combined an *in silico* analysis and literature data analysis to evaluate the genotype-dependent basal and *ex-vivo* stimulated expression level. Since the majority of common SNPs in the immune genes are located in promoter regions and thus may influence expression level, we provided an *in silico* analysis of allele-specific binding affinity of TFs. With the use of database information, we also explored linkage disequilibrium structure along with functional annotations of the SNPs within the genes under study.

## Results

### Genetic association studies of CAP and NP

Data with characteristics of the study populations are given in Supplementary Table S1. In total, the CAP study included 593 subjects, while the NP study comprised 571 patients at high risk of pneumonia development. In both studies, patients and controls were matched by age, sex and ethnicity. Genotyping data are presented in Supplementary Table S2. SNP frequencies were in Hardy–Weinberg equilibrium (HWE) in both control groups with an exception for *TLR4* rs4986791 (CAP controls). Several associations revealed in CAP and NP studies became non-significant after correction for multiple comparisons. However one observation is noteworthy. In the recent meta-analysis^6^ assessing the role of host genetic variations in susceptibility to several respiratory infectious diseases (tuberculosis, influenza, respiratory syncytial virus, SARS-Coronavirus and pneumonia), the *IL4* gene allele 2070874-T was the only variant associated with respiratory infections in the pooled group (tuberculosis and respiratory syncytial virus). The *IL4* rs2243250-T allele which is in complete linkage disequilibrium with the rs2070874-T allele (r^2^ = 1.0) was a susceptibility allele for NP in our study (Supplementary Table S2).

### Meta-analysis

#### Studies Characteristics

A total of 346 studies were identified by searching PubMed, Embase and Web of Knowledge resources (Supplementary Figure S1). The characteristics of the included studies are given in Supplementary Table S3. Ten studies were about associations of the selected genes with CAP, six papers were about HAP, five investigations considered pneumococcal disease, two studies were on Legionnaires’ disease and the rest one dealt with an unspecified type of pneumonia. HAP studies comprised the most heterogeneous group of observations. Sixteen studies indicated infectious pathogens (Supplementary Table S4), among them, five researches considered viral pneumonia. Deviations for Hardy-Weinberg equilibrium in controls were reveled mainly in the studies of the *IL10* SNPs (two works for each of the three considered polymorphic sites). Eighteen studies were performed in European population, two studies were conducted in Asian population, and three papers analyzed mixed population. All but four investigations were implemented in adult populations. Addressing pneumonia critical complications, the only available type of similar data in different studies was revealed for severe sepsis/septic shock in CAP/pneumococcal disease. In the analysis of this type of poor outcome, we also included our own results and data from the study of systemic inflammatory response syndrome (SIRS) in CAP patients (SIRS IV vs. SIRS I+II)^14^.

#### Quantitative data synthesis

**Figure 1:**
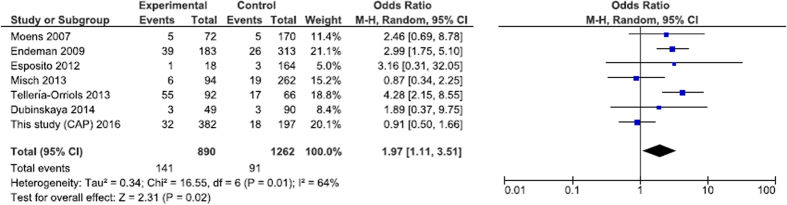
Forest plot for stratified analysis of the association between the *TLR2* (rs5743708) polymorphism and CAP/Legionnaires’ disease/pneumococcal disease (dominant model).

**Figure 2:**
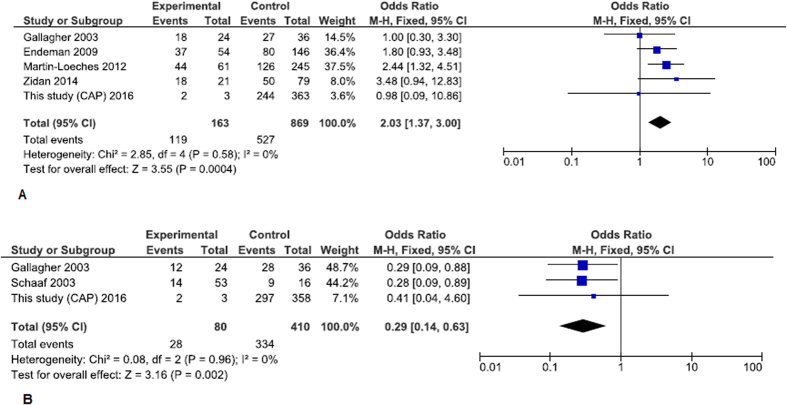
(**A**) Forest plot for stratified analysis of the association between the *IL6* (rs1800795) polymorphism and severe sepsis/septic shock/severe SIRS in CAP patients (dominant model); (**B**) Forest plot for stratified analysis of the association between the *IL10* (rs1800896) polymorphism and severe sepsis/septic shock/severe SIRS in patients with CAP/pneumococcal disease (dominant model). CAP, community-acquired pneumonia; SIRS, Systemic inflammatory response (score IV vs. score I+II).

We performed a total of eleven pneumonia susceptibility meta-analyses in four genetic models (Table 1) with no significant results for the whole data sets. A subset analysis after removing studies that were not consistent with HWE equation revealed a borderline significance for the association of the *IL10* rs1800896 GA/AA genotype with susceptibility to pneumonia/pneumococcal disease (Table 1). Further meta-analyses were stratified by pneumonia type and ethnicity. In the article of Yuan *et al*.^15^ there were no data on ethnicity and a portion of patients with pneumonia in pneumococcal disease. After excluding the study of Yang *et al*.^15^ and three HAP studies, *TLR2* rs5743708 minor genotype appeared to be associated with CAP/Legionnaires’ disease/pneumococcal disease (Fig. 1). Sensitivity analysis showed that these results were not stable (Supplementary Table S5). An analysis for severe sepsis/septic shock/SIRS in CAP/pneumococcal disease was available only for the SNPs *IL6* rs1800795 and *IL10* rs1800896 with both series providing significant results (Fig. 2). The so-called “low expression” allele -174C (rs1800795) of pro-inflammatory cytokine *IL6* was more frequent in patients with poor outcome (dominant model), while the so-called “low expression” allele -1082A (rs1800896) of anti-inflammatory cytokine *IL10* protected against the development of severe critical conditions (dominant model).

#### Correlation between SNP genotypes and gene expression

Classification of cytokine genotypes as high or low cytokine producers is often based on a relatively small number of initial publications. These papers primarily presented expression data in combination with genotyping data in healthy controls, various patient groups and under different stimulation protocols^16^. Over time, the results of these investigations were not always replicated; moreover some papers declared an opposite direction of association.

**Figure 3:**
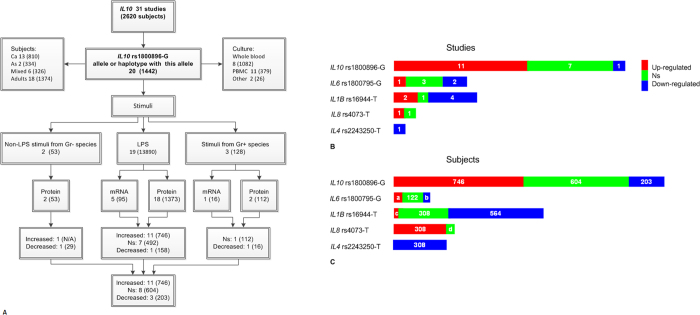
Summary of literature data on correlation between cytokine genotypes and *ex-vivo* stimulated expression level. (**A**) Block diagram for *IL10* investigations; (**B**) Proportions of studies that have found genotype-linked LPS-induced up-regulation, down-regulation or non-significant associations. (**C**) Total number of subjects in the above studies.

In this part of our investigation, we present an overview of studies comparing induced allele-specific cytokine expression profile. A total of 3612 studies were found on this theme by searching PubMed, Embase and Web of Knowledge resources (Supplementary Figure S2). It was assumed that (i) different stimuli may be more or less specialized in their allele-specific influence on cytokine production; (ii) patients with different diseases may have pre-existing conditions that multi-directionally affect cytokine level. Taking into account these considerations, we limited a spectrum of analyzed studies by the use of pneumonia related pathogen stimuli and considered samples only from healthy volunteers. Additional treatment, if applied, was also indicated to compare results for different stimuli in the same studies. Data from genetic association studies of interleukin genes polymorphisms and *ex vivo* response to bacterial toxins are collected in Supplementary Table S6. Studies differed by population ethnicity, polymorphic sites, cellular sources, and *in vitro* incubation protocols. Several studies measured mRNA, but the majority assessed protein secretion. Due to high variability, we could not perform quantitative data synthesis and present here the results of qualitative analysis. Summary for *IL10* investigations is given in a block diagram (Fig. 3a). The majority of studies have measured lipopolysaccharides (LPS)-stimulated IL10 production in association with *IL10* -1082G/A (rs1800896) genotype or haplotypes including -1082 alleles and testified that *IL10* -1082G allele may be a high producing allele. Since LPS is released from Gram-negative bacteria, the results should be extrapolated on Gram-positive species (e.g. *Streptococcus pneumonia*, *Staphylococcus aureus*) with caution. Proportions of data with genotype-linked LPS-induced up-regulation, down-regulation or non-significant associations for all studied cytokines are shown in Fig. 3b,c. Proportions are given for the number of studies (Fig. 3b) and for the number of subjects in these studies (Fig. 3c). Data for cytokines other than *IL10* could not be interpreted due to a small number of studies/subjects and contradictory associations. Similar analysis was not performed for toll-like receptor genes because genetic variations in these genes were mainly studied in association with cytokine expression.

**Figure 4:**
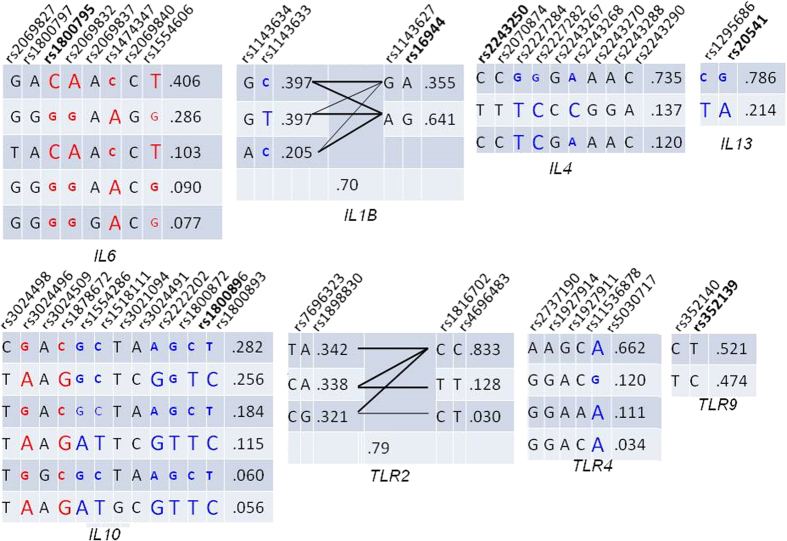
SNPexp data for correlation between SNP-based haplotype markers of the studied genes and mRNA expression level under basal conditions. Data were obtained from the HapMap phase II release 23 data for EBV-transformed lymphoblastoid cell lines of 60 CEU (Utah HapMap population) parents. Only haplotypes with frequencies more than 3% were included. Differences in gene expression in at least one from a series of available transcripts were considered. SNPs under study are in bold. Small letters for nucleotides correspond to a decreased expression level, while large letters represent an increased expression level. Dark blue color indicates a tendency level of association (P-value in a range 0.05–0.10); red color shows associations with P < 0.05 under additive model. The *TLR2* and *IL1B* genes include two linkage blocks, while other genes consist of one linkage block. Hedrick’s multiallelic D′, which represents the degree of LD between the two blocks when treating each haplotype within a block as an “allele” of that region are shown for the *TLR2* and *IL1B* genes. No haplotype markers were identified in the *IL8* gene.

Further we performed an *in silico* analysis of correlation between interleukin and toll-like receptor genotypes and basal gene expression levels utilizing SNPexp resource^17^. We constructed haplotypes with the HaploView software and analyzed association between check SNPs in haplotype blocks and mRNA expression levels in lymphoblastoid cell lines in the Utah HapMap population (CEU). Summary data are provided in Fig. 4. Weak association trends (0.05 < *P* < 0.10 for at least one SNP in the gene) were seen for *IL1B, IL4, IL13* and *TLR4* genes (Supplementary Table S7). Significant but also weak associations (*P* - value in the range 0.02–0.045) were revealed for *IL6* and *IL10* SNPs (Supplementary Table S7). These weak correlations cannot substantially contribute to differences in genotype-dependent expression of cytokines in acute infection, since cytokine production in these conditions is described in terms of fold-change.

#### SNPs influence on transcription factors’ binding affinity

**Figure 5:**
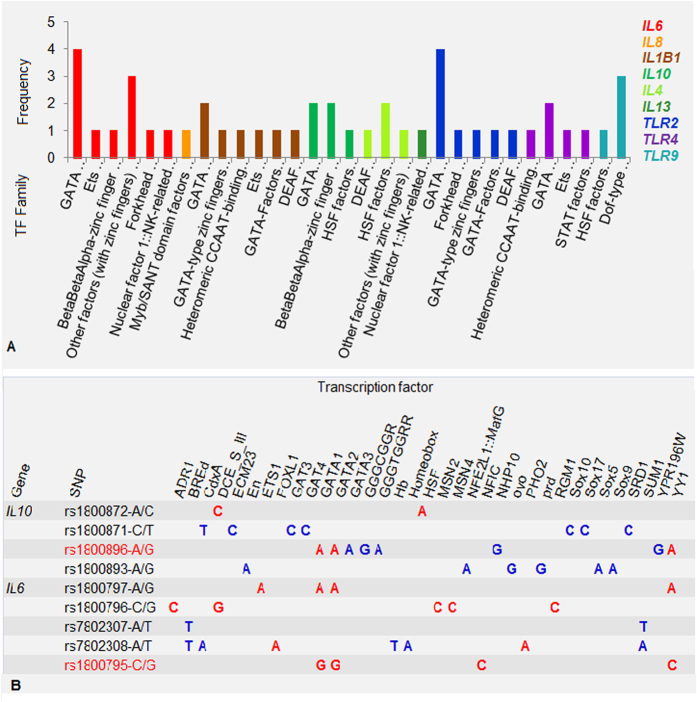
RegSNP results for transcription factor DNA-binding specificity for two alleles at each SNP in putative promoter regions of the studied genes. (**A**) By gene distribution of TFs grouped into families. The y-axis shows the frequency of SNPs influencing specific transcription factor DNA-binding ability of TF family members. The x-axis presents TF families. (**B**) An influence of different alleles within putative promoter regions of the *IL6* and *IL10* genes on binding affinity of TFs. SNPs under study are signed in red. Dark blue color indicates the minimum score difference cut-off in the range of 0.10 to 0.20; red color marks the minimum score difference cut-off >0.20.

One of the main controlled stages of gene expression is regulation of transcription, which largely depends on recruitment of transcription factors (TFs). Binding affinity of TFs may be affected by SNPs that fall in transcription factor binding sites. In this part of our study, we assessed an influence of different alleles within putative promoter regions of the studied genes on binding affinity of TFs. Twenty seven SNPs including studied SNPs (*IL10* rs1800896, *IL6* rs1800795, *IL1B* rs16944, *IL8* rs4073, *IL4* rs2243250, *TLR9* rs5743836 and rs187084) were identified in the putative promoter regions of the studied genes (2 kb upstream of a transcription start site). An *in silico* analysis of transcription factor DNA-binding specificity for two alleles at each SNP was conducted with RegSNP resource (http://viis.abdn.ac.uk/regsnp/Home.aspx) (Supplementary Table S8). Differences in allele-specific transcription factor DNA-binding ability in the whole set of twenty seven SNPs were predicted for in total 94 TFs. By gene distribution of TFs grouped into families (with the stringent cut-off level 0.20 for the minimum score difference) is shown in Fig. 5a.

In more detail, we present the analysis of the SNPs within putative promoter regions of the *IL6* and *IL10* genes (Fig. 5b). With the minimum score difference cut-off 0.10, binding affinity of 23 and 22 TFs was predicted to be influenced by four *IL10* and five *IL6* SNPs respectively. The *IL6* SNPs rs1800795 and rs1800797 (r^2^ = 0.97) affect binding affinity of GATA1, GATA2 and YYI TFs. The linked alleles rs1800795-G and rs1800797-G do influence on binding affinity of GATA1 and GATA2 factors in opposite directions (rs1800795-G has higher binding specificity, rs1800797-G has lower binding specificity), while the influence on transcription factor YYI affinity is unidirectional. We also used RegSNP resource for an analysis of transcription factors NF-1and Sp1 known to have a higher affinity for the rs1800795-C and rs1800896-G alleles respectively^18^,^19^. These factors were not covered by the initial analysis due to the stringent cut-off settings (see please Materials and Methods). RegSNP results for these TFs as well as for the above mentioned GATA1 factor (higher affinity for the rs1800795-G allele^20^) were in line with literature data (Supplementary Table S8, Sheets 1 and 2). Finally, we performed an analysis of the nuclear factor NF-kB, which was shown to be induced by a majority of pneumonia-related pathogens (http://www.bu.edu/nf-kb/gene-resources/target-genes/). The *IL10* SNP rs1800795 falls into low-affinity binding site for NF-kB, while the *IL6* SNP rs1800896 exerts some influence on NF-kB sequence-specific affinity (allele C higher) (Supplementary Table S8, Sheets 1 and 2).

The analysis of transcription factors’ affinity changes caused by SNPs yielded several noteworthy observations. First, alleles within one SNP could have increased or decreased affinity to different TFs. Second, GATA1-3 factors were highly overrepresented in our series: five of nine genes have SNPs that may influence GATA factors’ binding affinity. Moreover, all SNPs with potential GATA factors binding sites have predicted allele binding score differences >0.20. Third, in the above analysis of the *IL6* and *IL10* SNPs we showed that SNPs within the same putative promoter region may change the protein-DNA binding affinity of a specific TF /TF family member. This observation was supported by data analysis of the other SNPs (e.g. SOX-related factors and *IL10* SNPs; DCE SIII factor, Msn2/Msn4 factors and *IL13* SNPs; CCAAT/enhancer-binding proteins (C/EBPdelta and C/EBPbeta), HSF and *IL4* SNPs; GATA family members and *TLR2* SNPs (Supplementary Table S8).

#### Linkage disequilibrium and functional analysis of the selected genes

**Figure 6:**
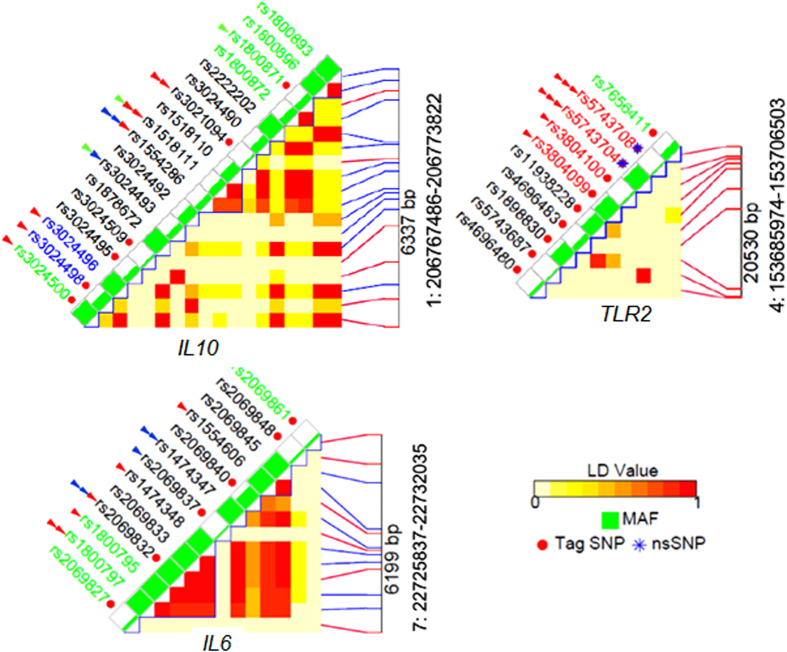
Linkage disequilibrium plots of the *IL6*, *IL10* and *TLR2* gene regions generated with the LD tag SNP selection tool within SNPinfo resource. Pair-wise LD values are indicated by different color, which changes from red to white as the D’ value decreases. SNP name is colored with genomic context: black, intron region; red: coding region; blue, UTR (untranslated) region, green, non-genic region. Minor allele frequency for each SNP in European population is denoted by the height of green bar. Functional annotations from the SNP info web server are shown with red arrows: one arrow, regulatory potential is in the range of 0.10–0.20; two arrows, regulatory potential is in the range of 0.20–0.30; three arrows, regulatory potential is higher than 0.30. Functional annotations from the RegulomeDB info web server are represented with dark blue arrows: one arrow, score 2a–f; two arrows, score 1a–f. Scores 3–7 are not provided. SNPs with category 7 score lack evidence of regulatory function, while category 1 variants are assumed to affect binding and expression of a gene target. Within subcategories a–f, variant scored as 1a has the highest confidence on functionality. Functional annotations from the NHGRI GWAS Catalog are marked by green arrows. The number of arrows corresponds to the number of the associations in the Catalog.

To systematically investigate functional characteristics of the SNPs within the selected genes we constructed LD blocks in European population and collected data on SNPs in these blocks utilizing three web-based resources: RegulomeDB^21^, SNPinfo^22^ and the NHGRI GWAS Catalog^23^ (Supplementary Table S9). *IL6*, *IL10* and *TLR2* genes are of the greatest interest in regard with the results of our meta-analysis. Data for these genes are given in Fig. 6, while characteristics of the remaining genes are provided in Supplementary Figure S3. *IL6* SNPs are in high LD; SNPs in putative promoter region, including rs1800795 have some regulatory potential, however the highest *in silico* functionality is predicted for the intron SNP rs2069832 which is adjacent to rs1800795 (distance 788 bp). *IL10* SNPs are in weak LD; putative promoter SNPs including rs1800896 have little if any regulatory potential. Three *IL10* SNPs were associated with diseases in the NHGRI GWAS Catalog (Supplementary Table S9). Several intron and 3’downstream SNPs that are not linked with the SNP rs1800896 may be more functional than the latter one. An analysis of LD blocks and functional annotations of the *IL6* SNPs shows that biological effects of these SNPs may be substantially influenced by the effects of proxy SNPs, while for the *IL10* SNPs the influence of neighboring SNPs is not profound. The *TLR2* functional variations are SNPs in coding regions, especially non-synonymous SNPs with known deleterious effects due to amino-acid change. Among SNPs in the other genes under study (Supplementary Figure S3), functional SNPs are located predominantly in the *IL1B*, *IL13* and *TLR9* gene; two SNPs in the *IL13* gene and one SNP in the *TLR4* gene have been associated with several disorders and traits in the NHGRI GWAS Catalog (Supplementary Table S9).

## Discussion

In this study, we investigated a role of host genetics in pneumonia development and progression. Quality of a genetic association study depends on many factors, with sample size being among the most important among them. In addressing gene candidate studies of pneumonia, the largest sample size across all the studies was used by Martin-Loeches *et al*.^13^ followed by our CAP and HAP groups. Nevertheless, we did not obtain results significant after correction for multiple comparisons. This is a rather typical situation, since common polymorphic variants have low penetrance and cannot provide strong association effects, meanwhile, more sites are analyzed the less probability that results will remain significant after testing for multiplicity. In view of this, the revealed association of the *IL4* rs2243250-T allele with NP, which is in line with the results of the recent meta-analysis (see please the Results section), seems worthwhile. Although IL4 is usually considered in regard to the immunoglobulin (Ig)-mediated allergic/inflammatory pathway, it is also known to influence humoral response during bacterial infection^24^ and suppress production of bacterial toxins-induced synthesis of proinflammatory cytokines^25^. IL4 affects pulmonary surfactant homeostasis^26^ and may be an important player in pulmonary clearance of bacteria and virus^27^. Given the results of allele-specific expression study^28^ together with the results of the meta-analysis of respiratory tract infectious diseases^6^ and data from our study, the rs2243250-T allele may be a genetic marker or a susceptibility allele in several respiratory infections.

Currently, there are a limited number of pneumonia-related meta-analyses of genetic association studies. In contrary to the majority of other common diseases, which are often subjected to numerous and repetitive meta-analyses, pneumonia meta-analyses considered a narrow range of genes and have never been updated (with the exception for the *IL6* association with pneumonia) (https://phgkb.cdc.gov/HuGENavigator/phenoPedia.do). Our study is the first to summarize data from individual papers on the role of *TLR2* SNPs in pneumonia/pneumococcal infection. Among TLRs, TLR2 is a receptor with the widest range of recognized pathogen-associated molecular patterns (PAMPs) including PAMPs from Gram-positive bacteria^29^. There was no association of *TLR2* with pneumonia in the whole set of meta-analyzed studies. Given that HAP is an extremely heterogeneous disease, we performed a stratified meta-analysis of CAP/Legionnaires’ disease/pneumococcal disease studies. Minor genotype *TLR2* rs5743708 GA/AA was associated with infection in this subset. To access an influence of amino-acid change (753 Arg > Gln) on enzyme structure and activity we utilized the HOPE resource^30^. The mutant residue differs from the wild-type one by size, charge and hydrophobic properties; multimer contacts peculiar to wild-type protein may be blocked in the mutant protein. To identify numerous PAMPs, TLR2 forms either homodimers or heterodimers with TLR1 or TLR6. The *TLR2* rs5743708 G to A transition leads to a functional deficiency in heterodimerization with TLR6 with subsequent diminished activation of intracellular signaling pathways^31^. This change in *TLR2* function correlates with the observed influence of this SNP on susceptibility to infections^6^,^32^.

Other noticeable results of our meta-analyses relate to the development of severe complications in pneumonia/pneumococcal infection. Severe sepsis/septic shock were associated with the *IL6* rs1800795 GC-CC and *IL10* rs1800896 G/G genotypes. The result for *IL6* was observed on the relatively large sample therefore it seems more reliable than the summary estimate for *IL10* based on the smaller sample size. Data for the *IL6* genotype GC-CC were also supported by its association with further worsening of critical illness from severe sepsis to septic shock in pneumonia patients^33^,^34^. IL6 is a multi-functional cytokine with both pro- and anti-inflammatory properties and a central role in host defence^35^. IL10 is the most potent anti-immune and anti-inflammatory cytokine. In infection, it provides a balance between immunosuppression and immunostimulation to maintain a homeostatic state^36^. Since both cytokines are key players in orchestrating immune response, we assume that alleles that protected against severe complications in pneumonia patients (the *IL6* rs1800795-G and *IL10* rs1800896-A) may provide more effective regulation in the cytokine network in specific pathophysiological conditions.

An assay for *ex vivo* cytokine stimulation is often applied to study genetically mediated interindividual variations of cytokine production upon specific stimuli. The results of such an assay provide data for classification of common polymorphic variants as “low” or “high” production ones. *Ex vivo* stimulated secretion of cytokines is considered to be more standardized assay than *in vivo* measurement of cytokine level. *Ex vivo* spontaneous cytokine production was declared not to occur or occur at low levels albeit with some exceptions^37^. Hence, in genetic association studies, the results of the *ex vivo* stimulation can be interpreted in terms of more or less inducibility for the carriers of different genotypes. There are many methodical circumstances influencing stimulated expression level, the most important of them, *in vivo* pre-existent conditions, the use of PBMCs vs. whole blood, the length of the incubation period, single or several concentrations of the stimuli applied and even the origin of this stimuli^16^,^37^,^38^. In general, literature data support an existing classification exclusively for the *IL10* “high” and “low” producing genotypes but only in certain cell types and in terms of LPS-induced *ex-vivo* IL10 secretion. Utilizing the SNPexp resource we showed that basal mRNA expression level was weakly affected by genetic background.

The pattern of cytokine responses to infection pathogens *in vivo* is thought to be much more complex than in model experiments. Age, sex, socio-economic status and even more so chronic disorders affect cytokine level^20^,^37^. Nevertheless, given the results of the meta-analyses it is interesting to consider *IL6* and *IL10* genotype-dependent expression data in pneumonia or conditions which may precede or follow pneumonia. In CAP studies, there were no significant associations between the *IL6* rs1800795 genotype and IL6 production in adult^39^,^40^ and pediatric cases^41^. High IL6 secretion was associated with the -174G allele in patients with severe sepsis^34^ and after cardiopulmonary bypass (CPB)^42^. In three other studies in CPB patients, the -174C allele has appeared to be high producing allele^43^,^44^,^45^. IL10 protein level has been not affected by *IL10* SNPs in three studies of CAP^39^,^40^,^46^ and one study of NP^47^. Significant but opposite results have been shown for -1082 G/G genotype associated with lower production of IL10 after CPB^48^ and with higher secretion of IL10 in burned patients^49^. Two papers have revealed the significant associations of a “high”- producing haplotype -1082G-819C-592C with higher level of *IL10* in adult and pediatric patients after CPB^50^,^51^. This brief survey demonstrated that *in vivo* genetic associations of IL6 and IL10 expression levels were rather contradictory.

Functional common SNPs in cytokine genes are mainly located in putative promoter regions. They may fall inside TFBS and alter transcription factor DNA-binding ability. We explored 2 kbp 5′-upstrem gene region of the studied genes for common SNPs and analyzed their sequence-specific influence on TF binding affinity. Our analysis showed that GATA1-3 factors were highly overrepresented in the set. GATA1-3 factors play a pivotal role in hematopoiesis but also are known to be involved in innate and adaptive immunity. They regulate immune response against different pathogens including those that may cause pneumonia^52^. Among other common TFs there were HSFs (heat-shock factors). SNPs that influence binding affinity of HSFs were located in anti-inflammatory cytokines *IL4* (two SNPs), *IL10* and in toll-lire receptor genes *TLR4, TLR9*. Functional activity of these SNPs may be mediated by HSFs’ dependent activation of the anti-inflammatory limb of the immune response^53^. We also demonstrated that TF family members have similar allele-specific binding affinity (e.g. GATA factors, SOX-related factors) and linked alleles of SNPs may influence binding affinity of the same TF uni or multi-directionally. Clustering of TFBS for the same TF (homotypic clusters of TFBSs (HCTs)) is known to be enriched in promoters. It has been suggested that HCTs provide functional advantages being favorable for recruitment of TFs^54^.

It is known that the same TFs can repress or activate transcription depending on cell type and specific promoter sequence. There can be different coexisting modes of TF action: direct activation or repression, indirect activation or repression via interaction with cofactors, induction or suppression by disruption of binding sites and other^55^. Given complex and optional interactions of TFs with TFBS, our *in silico* analysis was not either definitive or exhaustive but rather tentative and suggestive with an intension to test the approach for further more detailed investigations supported by experimental data.

Combining LD analysis with functional information data, we showed that a relatively higher overlap with functional annotations was found for the *IL6* and *IL10* SNPs. We did not reveal predominance of functional SNPs in certain regions of the all studied genes; they were more or less evenly distributed within genes. The *TLR2* and *TLR4* genes size was several times larger than the cytokine genes size. High LD was evident for the *IL6*, *IL4* and *IL8* genes while low LD was detected for the *TLR2* and *TLR4* genes. This means that the estimated genetic associations reflect non-independent effects of tightly linked SNPs in the *IL6* and *IL4* genes. The *TLR2* SNP rs5743708 is not linked with the other functional *TLR2* SNP rs5743704 hence the amino-acid change 753Arg > Gln (rs5743708) may solely contribute to the revealed association of the *TLR2* gene with pneumonia.

The study has serious limitations. Own experimental data includes only genotyping of the SNPs in six cytokine genes and three genes of toll-like receptors among CAP and HAP subjects. CAP and HAP samples were modest and the study was powered to detect only relatively large effect sizes (minimum detectable OR~1.6–1.9). Our patients were mainly men and the results may not be generalizable to women. Genes with an important role in the development and output of inflammation such as *TNFa, IL1R, LTA, TGFb* were not included in the analysis. Highly heterogeneous spectrum of pneumonia-related infections in adult and pediatric populations with very different pathogens with different virulence and different sensitivity to antibiotics were considered. The analysis of SNPs influence on DNA - TF binding affinity was limited by the stringent cut-off settings. However, the study has some biological and methodological implications. We combined own experimental data and the results of the meta-analyses with the analyses of the totality of published data (expression studies) and web-based resources. Our original analysis of transcription factors’ affinity changes caused by SNPs did not and could not provide an unequivocal interpretation in terms of “low” and “high” allele-specific expression phenotypes. Instead *in silico* obtained data demonstrated complexity in an assessment functionality of SNPs that fall inside TFBS. Consideration of the enrichment for the functional SNPs in LD blocks may help to estimate contribution of the individual SNPs in the observed or presumed associations.

In conclusion, the current study presents some experimental data and the results of the meta-analyses related to the role of host genetics in pneumonia development and progression. Other results were obtained from literature and web-based resources. Since the last data have no experimental support, care must be taken with our tentative and hypothesis generating findings, which require testing in further investigations.

## Materials and Methods

### CAP and HAP case-control studies

The study protocol was approved by the Ethics Committee of the V. A. Negovsky Research Institute of General Reanimatology (with IRB approval number 2/6/2012), and adhered to the tenets of the Declaration of Helsinki. Informed consent was obtained from all subjects.

From January 2008 to February 2016, CAP and HAP studies were conducted at the hospitals of the V.A. Negovsky Research Institute of General Reanimatology, Moscow, Russia. Three hundred ninety CAP patients were recruited in the study; the corresponding control group consisted of 203 unrelated healthy volunteers without a previous history of pneumonia. Subjects with severe physical trauma due to the accidents and patients with acute diseases requiring extensive surgery were included in the prospective study of NP; among 571 patients, 355 individuals developed NP. The majority of patients and controls in both studies were workers of the Rescue Service, predominantly practically healthy young or middle-aged men. The current study is a continuation of previously published CAP and NP investigations hence inclusion/exclusion criteria, microbiological procedures, genotyping methods and statistical procedures are available elsewhere^56^,^57^.

### Meta-analysis

A final search was performed on 4^th^ April 2016 of the PubMed, EMBASE, and Web of Science databases. We used the following keyword terms as the criteria for searching: pneumonia, pneumonia outcome, pneumonia severity, sepsis, septic shock, systemic inflammatory response, Streptococcus pneumonia, pneumococcus and gene polymorphism, genotype, alleles, variants and interleukin 6, interleukin-6, *IL6*, *IL-6* or interleukin 10, interleukin-10, *IL10*, *IL-10,* or interleukin 8, interleukin-8, *IL8*, *IL-8,* or interleukin 1B, interleukin-1B, *IL1B*, *IL-1B,* or interleukin 4, interleukin-4, *IL4*, *IL-4,* or interleukin 13, interleukin-13, *IL13*, *IL-13,* or toll-like receptor 2, *TLR2*, or toll-like receptor 4, *TLR4*, or toll-like receptor 9, *TLR9.* Two investigators (LS and SS) independently extracted data and reached consensus on all the items. We used the following inclusion criteria. The study had to be published in English or in Russian. The article must have evaluated prevalence rates of any type of pneumonia or pneumococcal disease in connection with genotyping data. To be included in the meta-analysis, the studies should provide enough data to calculate odds ratio (OR) with its 95% confidence interval. Additionally we searched for the studies assessing the association of the studied polymorphic variants with severe sepsis/septic shock or systemic inflammatory response (SIRS) in patients with pneumonia. The differences between sepsis and SIRS are that in sepsis individuals meet criteria for SIRS and have a known infection. Patients with SIRS and acute organ dysfunction may be termed patients with severe SIRS (score IV) (https://en.wikipedia.org/wiki/Systemic_inflammatory_response_syndrome#cite_note-rippe-3). In this context, we combined patients with SIRS IV and severe sepsis/septic shock into a single group with the most severe pneumonia-related critical conditions.

Statistical analysis was conducted with the Review Manager 5.3 software^58^. Pooled effects were calculated under the following genetic models (additive, dominant, recessive and overdominant). The Hardy-Weinberg equilibrium was assessed using Pearson’s χ2 test. The heterogeneity index I^2^ (I-squared) was used to assess the between-study heterogeneity. Values of I^2^ in the range of 0–25% were considered indicating negligible heterogeneity with the fixed-effect model applied, 25–50% – modest heterogeneity with both the fixed-effect and random-effect models applied, >50% – significant heterogeneity requiring the random-effect model. We conducted sensitivity analyses to explore the influence of individual studies on the pooled estimate. We did not assess publication bias for revealed associations because, when the number of studies is lower than ten, sensitivity of the tests is low^59^.

### Genotype-specific expression analysis

We collected literature data on genotype-specific *ex-vivo* induced expression level of the studied genes. We used the following keyword terms as the criteria for searching: expression, secretion, production and gene polymorphism, genotype, alleles, variants and interleukin 6, interleukin-6, *IL6*, *IL-6* or interleukin 10, interleukin-10, *IL10*, *IL-10,* or interleukin 8, interleukin-8, *IL8*, *IL-8,* or interleukin 1B, interleukin-1B, *IL1B*, *IL-1B,* or interleukin 4, interleukin-4, *IL4*, *IL-4,* or interleukin 13, interleukin-13, *IL13*, *IL-13.*In order not to miss related articles we did not use other terms and assorted papers after reading abstracts.

We performed an *in silico* analysis of the allele- specific mRNA expression level under basal conditions using SNPexp online tool^17^. We constructed haplotypes of SNPs in the studied genes in CEU population (Utah residents with ancestry from northern and western Europe) with the HaploView software (version 4.2). All within gene SNPs and SNPs in the 2000-bp upstream 5 flanking and the 500-bp downstream 3 flanking regions were included in the haplotype analysis. Haplotype blocks were defined using the method of confidence intervals^60^. Phased haplotypes from 210 individuals in the HapMap in correlation with expression data for 47294 transcripts in EBV transformed lymphoblastoid cell lines are available through the SNPexp database. By using web service linear regression testing we correlated mRNA expression level with genotypes in the 60 parents in CEU parent/parent/offspring trios^17^.

### Allele-specific transcription factor DNA-binding affinity analysis

We searched for the SNPs with MAF >0.10 within the putative promoter regions of the studied genes (2 kb upstream of a transcription start site) utilizing the NCBI Gene database. An *in silico* analysis of transcription factor binding site likelihood scores at positions of SNPs was performed with RegSNP resource (http://viis.abdn.ac.uk/regsnp/Home.aspx). In RegSNP web server, experimentally validated TFBS from the publicTransfac and the Jaspar databases are converted into a positional weight matrix. We used the following analysis options: species, human; cut-off value for minimum transcription factor binding site length, 4; cut-off value for minimum transcription factor likelihood score, 0.85; cut-off value for minimum score difference, 0.1; cut-off value for minimum score ratio, 0.1.

### Functional and linkage disequilibrium analysis

We explored LD patterns in the genes under study (including 2 kbp 5′ upstream and 500 bp 3′downstream regions) with the tool LD tag SNP selection within SNPinfo resource^22^. Since HapMap LD pairwise data do not include all the SNPs considered in our analysis, we used dbSNP genotype data in European population. We calculated pairwise LD values of all SNPs in the studied genes and plotted LD maps with the online program (http://snpinfo.niehs.nih.gov/cgi-bin/snpinfo/snpfunc.cgi)^22^. Functional analysis was performed with SNPinfo bioinformatic tool SNP Function Prediction (FuncPred; http://snpinfo.niehs.nih.gov/cgi-bin/snpinfo/snpfunc.cgi), RegulomeDB database^21^ and the NHGRI GWAS Catalog^23^.

## Additional Information

**How to cite this article**: Smelaya, T. V. *et al*. Genetic dissection of host immune response in pneumonia development and progression. *Sci. Rep.* **6**, 35021; doi: 10.1038/srep35021 (2016).

## Supplementary Material

Supplementary Dataset 1

Supplementary Table S8

## Figures and Tables

**Table 1:** Results of meta-analyses.

Gene, SNP	Disease	Studies (n)	Cases/Controls (n)	Genetic model^1^	I^2^ (%)	OR [95% CI]	P
*Il6* rs1800795	CAP/HAP/pneumococcal disease	7	2425/1879	Dom	6	0.92 [0.81–1.04]	0.20
	Subset (CAP)	5	1981/1623	Dom	2	0.88 [0.77–1.01]	0.08
	Subset (Pneumococcal CAP/pneumococcal disease)	4	1122/3034	Add	94	1.44 [0.71–2.95]	0.32
*IL1B* rs16944	CAP/HAP	4	797/558	Dom	35	0.88 [0.70–1.11]	0.30
*IL8* rs4073	CAP/HAP	4	2078/1654	Add	88	1.29 [0.87–1.90]	0.21
	Severe sepsis/septic shock in CAP	3	222/1444	Add	3	1.15 [0.82–1.62]	0.41
*IL10* rs1800896	CAP/HAP/pneumococcal disease	6	1167/885	Rec	72	1.41 [0.89–2.22]	0.14
	Subset (CAP)	4	752/625	Rec	82	1.68 [0.81–3.49]	0.16
	Subset (controls are in HWE)	4	1033/789	Rec	0	**1.26 [1.00**–**1.59]**	**0.05**
*Il10* rs1800871	CAP/Postoperative pneumonia in patients with esophageal cancer	3	188/194	Dom	84	1.44 [0.38–5.41]	0.59
*IL10* rs1800872	CAP/Pneumonia complicated with sepsis/Pneumonia in kidney transplant recipients	5	566/884	Dom	80	0.63 [0.32–1.24]	0.18
	Subset (controls are in HWE)	3	686/892	Add	8	0.86 [0.68–1.09]	0.21
*TLR2* rs5743708	CAP/Legionnaires’ disease/pneumococcal disease/HAP/pneumonia after allogeneic hematopoietic stem cell transplantation/pneumonia in AML patients after induction chemotherapy	11	1460/2119	Dom	71	1.60 [0.93–2.75]	0.09
*TLR4* rs4986790	CAP/Legionnaires’ disease/pneumococcal disease/HAP/pneumonia after allogeneic hematopoietic stem cell transplantation/pneumonia in AML patients after induction chemotherapy	9	1149/1867	Dom	63	1.18 [0.75–1.86]	0.48
	Subset (CAP/Legionnaires’ disease/pneumococcal disease^a^)	6	926/913	Dom	52	1.59 [0.94–2.69]	0.09
*TLR4* rs4986791	CAP/Legionnaires’ disease/pneumococcal disease/HAP/VAP/pneumonia in AML patients after induction chemotherapy	7	1523/1355	Dom	73	0.90 [0.50–1.62]	0.73
	Subset (CAP/Legionnaires’ disease/pneumococcal disease)	3	885/457	Dom	59	1.31 [0.54–3.18]	0.56
*TLR9* rs187084	CAP/HAP/pneumonia after allogeneic hematopoietic stem cell transplantation	3	798/536	Dom	45	0.89 [0.70–1.14]	0.35
*TLR9* rs5743836	CAP/HAP/pneumonia after allogeneic hematopoietic stem cell transplantation	3	809/537	Dom	56	1.21 [0.81–1.83]	0.35

Genetic models: Add, additive model (major allele versus minor allele); Dom, dominant model (heterozygote-minor homozygote versus major homozygote); Rec, recessive model (minor homozygote versus major homozygote- heterozygote). Significant results are in bold. Abbreviations: AML, acute myeloid leukemia; CAP, community acquired pneumonia; HAP, hospital acquired pneumonia; VAP, ventilator associated pneumonia.

^a^Pneumococcal disease studies with the majority of patients diagnosed with pneumonia are considered.
