# Supplementary material for: Genetic dissection of host immune response in pneumonia development and progression
Source: Sci Rep. 2016 Oct 11;6:35021. doi: 10.1038/srep35021 (PMC5057148; doi:10.1038/srep35021)
Supplement: Supplementary Dataset 1 [file srep35021-s1.pdf]

Supplementary Data

Genetic dissection of host immune response in pneumonia development and progression

Tamara V. Smelaya, PhD, MD[a], Olesya B. Belopolskaya, PhD[b], Svetlana V. Smirnova[b], Artem N. Kuzovlev PhD, MD[a], Viktor V. Moroz, PhD, MD[a], Arkadiy M. Golubev, PhD, MD[a], Noel A. Pabalan (PhD)[c], Lyubov E. Salnikova, PhD, Dr. Sci. (Biol.)[a,b*]

[a]V. A. Negovsky Research Institute of General Reanimatology, Russian Academy of Sciences, 25 Petrovka str., build.2, Moscow 107031, Russia;

[b]N.I. Vavilov Institute of General Genetics, Russian Academy of Sciences, 3 Gubkin street, Moscow 117971, Russia;

[c]Center for Research and Development, Angeles University Foundation, Angeles City 2009, Philippines

Correspondence to: Lyubov E. Salnikova, Institute of General Genetics, Russian Academy of Sciences, 3 Gubkin Street, Moscow 117971, Russia, phone 74991328958, fax 4991328962

E-mail: salnikovalyubov@gmail.com

Supplementary Table S1.Characteristics of the patients included in the study

Characteristics	CAP		NP	
	Controls	Cases	Controls	Cases
	n (portion)<br>Mean ± SD		n (portion)<br>Mean ± SD	
Total number	203	390	216	355
Age (year)	29.66 ± 10.99	28.41 ± 16.27	45.65 ± 17.16	44.44 ± 18.43
Male sex (n, %)	158 (0.78)	352 (0.90)	170 (0.79)	294 (0.83)
Pre-existing conditions				
• Cardiovascular diseases	-	0 (0.0)	14 (0.06)	31 (0.09)
• Diabetes	-	0 (0.0)	5 (0.02)	7 (0.02)
• Gastric/duodenal ulcer	-	1 (0.003)	15 (0.07)	12 (0.03)
• Neurological pathology	-	2 (0.005)	7 (0.03)	11 (0.03)
• Obesity	-	0 (0.0)	10 (0.05)	18 (0.05)
• Solid tumors (remission)	-	0 (0.0)	19 (0.09)	11 (0.03)
Infectious pathogens in BAL fluid[a]				
• Gram-positive bacilli	-	121 (0.31)	-	20 (0.06)
• Gram-negative bacilli	-	42 (0.11)	-	80 (0.23)
• Mixed Gram-negative and Gram-positive bacteria	-	70 (0.18)	-	29 (0.08)
ICU admission	-	184 (0.47)	-	303 (0.85)
ICU length of stay (days)	-	3.45 ± 3.61	-	17.46 ± 17.67
Patients on mechanical ventilator	-	9 (0.02)	32 (0.15)	125 (0.35)
APACHE II score[b]	-	10.9 ± 3.2	14.94 ± 3.95	15.54 ± 4.05
SOFA score[c]	-	-	5.54 ± 2.43	6.39 ± 2.61
Diagnosis at admission				
• CAP	-	390	0 (0.0)	0 (0.0)
• Severe combined trauma/wounding	-	0 (0.0)	121 (0.56)	186 (0.52)
• Bowel obstruction	-	0 (0.0)	17 (0.08)	23 (0.06)
• Inflammatory diseases of the abdominal cavity and retroperitoneal space complicated by destruction	-	0 (0.0)	42 (0.19)	106 (0.30)
• Purulent-inflammatory diseases of the skin, subcutaneous tissue	-	0 (0.0)	20 (0.09)	21 (0.06)
• Other	-	0 (0.0)	16 (0.07)	19 (0.05)
Critical conditions	-			
• ARDS		11 (0.03)	8 (0.04)	76 (0.21)
• Severe sepsis/septic shock[d]	-	3 (0.008)	27 (12.5)	49 (13.8)
Hospital mortality	-	6 (0.015)	52 (0.24)	95 (0.27)

[a]Microbiological data are specified in Supplementary Table S4.
[b]The severity of each patient was assessed with the use of the Acute Physiology and Chronic Health Evaluation (APACHE) II score within the first 24 h after ICU admission (Knaus et al. 1985).
[c]The Sequential Organ Failure Assessment (SOFA) score was evaluated as an indicator of organ dysfunction (Vincent et al. 1986).
[d]Sepsis, severe sepsis and septic shock were diagnosed according Surviving Sepsis Campaing 2012 (sccm.org›documents/SSC-Guidelines.pdf)

Abbreviations: ARDS, Acute respiratory distress syndrome; BAL, Bronchoalveolar lavage; CAP, Community-acquired pneumonia, ICU, Intensive care unit; NP, Nosocomial pneumonia; SD, Standard deviation

Supplementary Table S2. The distribution of genotypes among CAP and NP patients and corresponding controls

Genes and genotypes		Controls Number (%)	CAP cases Number (%)	P-value crude (genetic model), OR (95% CI)	P-value adjusted[a] (genetic model), OR (95% CI)	Controls Number (%)	NP cases Number (%)	P-value crude (genetic model), OR (95% CI)	P-value adjusted[b] (genetic model), OR (95% CI)
IL1B rs16944		n=199 HWE=0.77	n=383 **HWE=0.026**	0.16 (rec) 0.68 (0.40 – 1.16)	0.13 (rec) 0.65 (0.38 – 1.12)	n=210 HWE=0.29	n=347 HWE=0.86	**0.024 (dom) 0.67 (0.47 – 0.95)**	**0.019 (dom) 0.64 (0.44 – 0.93)**
	G/G	82 (41.2)	152 (39.7)			80 (38.1)	166 (47.8)		
	G/A	90 (45.2)	194 (50.6)			105 (50.0)	147 (42.4)		
	A/A	27 (13.6)	37 (9.7)			25 (11.9)	34 (9.8)		
IL8 rs4073		n=202 HWE=0.45	n=385 HWE=0.87	0.058 (dom) 1.43 (0.99 – 2.08)	**0.039 (add) 1.30 (1.01 – 1.67)**	n=208 HWE=0.20	n=346 HWE=0.34	0.44 (dom) 0.86 (0.59 – 1.26)	0.58 (dom) 0.89 (0.60 – 1.33)
	T/T	67 (33.2)	99 (25.7)			59 (28.4)	109 (31.5)		
	T/A	94 (46.5)	194 (50.4)			112 (53.9)	178 (51.5)		
	A/A	41 (20.3)	92 (23.9)			37 (17.8)	59 (17.1)		
IL6 rs1800795[c]		n=203 HWE=0.46	n=389 HWE=0.28	0.25 (dom) 0.81 (0.56 – 1.17)	0.32 (dom) 0.82 (0.56 – 1.21)	n=206 HWE=0.59	n=344 HWE=0.74	0.35 (add) 1.13 (0.88 – 1.45)	0.39 (add) 1.12 (0.86 – 1.46)
	G/G	57 (28.1)	127 (32.6)			68 (33.0)	104 (30.2)		
	G/C	106 (52.2)	186 (47.8)			104 (50.5)	173 (50.3)		
	C/C	40 (19.7)	76 (19.5)			34 (16.5)	67 (19.5)		
IL4 rs2243250		n=200 HWE=0.26	n=385 HWE=0.51	0.90 (dom) 0.98 (0.69 -1.38)	0.59 (dom) 0.91 (0.64 – 1.30)	n=209 HWE=0.53	n=347 **HWE=0.011**	**0.027 (dom) 1.48 (1.04 – 2.11)**	**0.017 (dom) 1.57 (1.08 – 2.29)**
	C/C	108 (54.0)	210 (54.5)			132 (63.2)	186 (53.6)		
	C/T	82 (41.0)	152 (39.5)			70 (33.5)	148 (42.6)		
	T/T	10 (5.0)	23 (6.0)			7 (3.4)	13 (3.8)		
IL10 rs1800896		n=199 HWE=0.35	n=384 **HWE=0.011**	0.064 (rec) 1.47 (0.98 – 2.22)	**0.038 (add) 1.33 (1.01 – 1.75)**	n=210 HWE=0.23	n=346 HWE=0.82	0.20 (rec) 1.32 (0.86 – 2.02)	0.26 (rec) 1.29 (0.82 – 2.01)
	G/G	50 (25.1)	71 (18.5)			57 (27.1)	89 (25.7)		
	G/A	106 (53.3)	216 (56.2)			113 (53.8)	175 (50.6)		
	A/A	43 (21.6)	97 (25.3)			40 (19.1)	82 (23.7)		
IL13 rs20541		n=201 HWE=0.07	n=386 HWE=0.95	0.36 (rec) 0.75 (0.41 – 1.38)	0.30 (rec) 0.72 (0.38 – 1.34)	n=209 HWE=0.92	n=347 HWE=0.76	0.50 (add) 0.91 (0.69 – 1.20)	0.44 (add) 0.89 (0.67 – 1.19)
	C/C	114 (56.7)	207 (53.6)			111 (53.1)	193 (55.6)		
	C/T	68 (33.8)	151 (39.1)			83 (39.7)	133 (38.3)		
	T/T	19 (9.4)	28 (7.2)			15 (7.2)	21 (6.0)		
TLR2 rs5743708		n=197 HWE=0.50	n=382 HWE=0.39	0.76 (dom) 0.91 (0.50 – 1.66)	0.56 (dom) 0.83 (0.44 – 1.55)	n=210 HWE=0.54	n=347 HWE=0.62	0.18 (dom) 0.62 (0.31 – 1.23)	0.18 (dom) 0.60 (0.29 – 1.25)
	G/G	179 (90.9)	350 (91.6)			193 (91.9)	329 (94.8)		

	G/A	18 (9.1)	32 (8.4)			17 (8.1)	18 (5.2)		
	A/A	0 (0.0)	0 (0.0)			0 (0.0)	0 (0.0)		
		n=196	*n*=382			n=210	n=345		
TLR2		HWE = 0.37	HWE = 0.63	0.47 (rec)	0.35 (rec)	HWE=0.67	HWE=0.23	0.21 (rec)	0.12 (rec)
rs4696480	T/T	51 (26%)	106 (27.8)	1.17 (0.77 – 1.77)	1.23 (0.79 – 1.91)	62 (29.5)	101 (29.3)	1.31 (0.86 – 1.19)	1.42 (0.91 – 2.23)
	T/A	104 (53.1)	186 (48.7)			107 (51.0)	161 (46.7)		
	A/A	41 (20.9)	90 (23.6)			41 (19.5)	83 (24.1)		
		n=191	n=372			n=209	n=343		
TLR4		**HWE=0.002**	HWE=0.43	0.39 (add)	0.54 (add)	HWE=0.36	HWE=0.22	**0.014 (add)**	**0.0032 (add)**
rs4986791	C/C	162 (84.8)	318 (85.5)	0.83 (0.54 – 1.28)	0.87 (0.55 – 1.36)	167 (79.9)	300 (87.5)	**0.56 (0.36 – 0.89)**	**0.48 (0.30 – 0.79)**
	C/T	24 (12.6)	53 (14.2)			41 (19.6)	43 (12.5)		
	T/T	5 (2.6)	1 (0.3)			1 (0.5)	0 (0.0)		
		n=196	n=382			n=210	n=347		
TLR9		HWE=0.066	HWE=0.96	0.21 (dom)	0.21 (dom)	HWE=0.78	HWE=0.49	0.42 (rec)	0.59 (rec)
rs352139	G/G	59 (30.1)	96 (25.2)	1.28 (0.87 – 1.88)	1.29 (0.87 – 1.92)	64 (30.5)	107 (30.8)	0.84 (0.54 – 1.29)	0.88 (0.55 – 1.40)
	G/A	85 (43.4)	191 (50.1)			102 (48.6)	177 (51.0)		
	A/A	52 (26.5)	94 (24.7)			44 (20.9)	63 (18.2)		
		n=196	n=382			n=208	n=343		
TLR9		HWE=0.64	HWE=0.89	0.18 (dom)	0.41 (dom)	HWE=0.55	HWE=0.72	0.76 (add)	0.91 (dom)
rs5743836	T/T	164 (83.7)	302 (79.1)	1.36 (0.86 – 2.13)	1.21 (0.76 – 1.93)	161 (77.4)	264 (77.0)	1.06 (0.73 – 1.54)	0.97 (0.63 – 1.50)
	T/C	30 (15.3)	75 (19.6)			45 (21.6)	73 (21.3)		
	C/C	2 (1.0)	5 (1.3)			2 (1.0)	6 (1.8)		
		n=194	n=368			n=207	n=340		
TLR9		HWE=0.79	HWE=0.70	0.56 (rec)	0.72 (rec)	HWE=0.68	HWE=0.72	0.40 (add)	0.35 (add)
rs187084	T/T	65 (33.5)	125 (34.0)	0.87 (0.55 – 1.37)	0.92 (0.57 – 1.47)	57 (27.5)	104 (30.6)	0.90 (0.70 – 1.15)	0.88 (0.68 – 1.15)
	T/C	93 (47.9)	182 (49.5)			106 (51.2)	171 (50.3)		
	C/C	36 (18.6)	61 (16.6)			44 (21.3)	65 (19.1)		

Significant *P*-values are in bold. The genetic model: add, additive; dom, dominant; rec, recessive. HWE, Hardy-Weinberg equilibrium P-value

[a]Adjusted by sex and age

[b]Adjusted by sex, age, APACHE II score and the use of mechanical ventilation more than 24 hours

[c]In our CAP study a total of 593 patients were analyzed, and 475 out of them had been previously analyzed for the *IL6* rs1800795; in NP study, from a total of 571 subjects, 321 subjects had been previously analyzed for the *IL6* rs1800795 (Salnikova et al. 2013).

Supplementary Table S3

Characteristics of meta-analyzed studies

First author	Year	Country	Cases	Controls[a]	Sample size Controls/ Cases	Controls			Cases			HWE in controls	Methods of genotyping	Quality[b]
Il6 rs1800795 (-174C>G)						GG	GC	CC	GG	GC	CC			
Schaaf	2005	Germany	Pneumococcal disease, adult	HB	50/100	17	25	8	29	51	20	0.81	SSP-PCR	CCB
Endeman	2011	Netherlands	CAP, adult	PB	311/200	113	150	48	83	92	25	0.88	Taqman	CCB
Martin-Loeches	2012	Spain	CAP, adult	PB	953/1246	438	413	102	581	516	130	0.75	Taqman	CCA
Martinez-Ocana	2013	Mexico	Viral CAP, adult	PB	46/65	39	7	0	53	12	0	0.58	PCR-dot blot	CCB
Zidan	2014	Egypt	CAP, pediatric	PB	110/100	22	60	28	32	55	13	0.32	PCR-RFLP	CCC
This study	2016	Russia	CAP, adult	PB	203/389	57	106	40	127	186	76	0.46	PCR-CTPP	CCA
This study	2016	Russia	HAP, adult	HB	206/344	68	104	34	104	173	67	0.59	PCR-CTPP	CCB
IL8 rs4073 (-251A>T)						TT	TA	AA	TT	TA	AA			
Endeman	2011	Netherlands	CAP, adult	PB	313/199	62	153	98	46	94	59	0.87	Taqman	CCB
Georgitsi	2016	Greece	CAP with sepsis, adult	NM	104/109	28	52	24	50	50	9	0.99	PCR-RFLP	CCB
This study	2016	Russia	CAP, adult	PB	202/385	67	94	41	99	194	92	0.45	PCR-CTPP	CCA
This study	2016	Russia	HAP, adult	HB	208/346	59	112	37	109	178	59	0.20	PCR-CTPP	CCB
IL1B rs16944 (-511T>C)						CC	CT	TT	CC	CT	TT			
Wan	2013	China	Pneumonia in kidney transplant recipients, adult	Kidney transplant recipients without pneumonia	63/33	14	30	19	7	16	10	0.74	PCR-RFLP	CCC
Sakamoto	2014	Japan	Postoperative pneumonia in patients with esophageal cancer, adult	Patients without postoperative pneumonia	86/34	26	60		8	26		NA	ARMS-PCR	CCC
This study	2016	Russia	CAP, adult	PB	199/383	82	90	27	152	194	37	0.77	PCR-CTPP	CCA
This study	2016	Russia	HAP, adult	HB	210/347	80	105	25	166	147	34	0.29	PCR-CTPP	CCB

Il10 rs1800896 (-1082A>G)						GG	GA	AA	GG	GA	AA			
Schaaf	2003	Germany	Pneumococcal disease[c], adult	HB	50/69	13	17	20	16	28	25	**0.03**	ARMS-PCR	CCC
Endeman	2011	Netherlands	CAP, adult	PB	313/200	74	170	69	54	90	56	0.13	Taqman	CCB
Martinez-Ocana	2013	Mexico	Viral CAP, adult	PB	46/65	9	32	5	7	22	36	**0.006**	PCR-dot blot	CCC
Romanova	2013	Russia	Viral pneumonia, adult	NM	67/103	29	30	8	78	17	8	0.96	Allele-specific PCR	CCC
This study	2016	Russia	CAP, adult	PB	199/384	50	106	43	71	216	97	0.35	PCR-CTPP	CCA
This study	2016	Russia	HAP, adult	HB	210/346	57	113	40	89	175	82	0.23	PCR-CTPP	CCB
Il10 rs1800871 (-819T>C)						TT	TC	CC	TT	TC	CC			
Martinez-Ocana	2013	Mexico	Viral CAP, adult	PB	46/65	12	34	0	7	55	3	**0.000**	PCR-dot blot	CCC
Romanova	2013	Russia	Viral pneumonia, adult	NM	62/89	13	9	40	8	19	62	**0.000**	Allele-specific PCR	CCC
Sakamoto	2014	Japan	Postoperative pneumonia in patients with esophageal cancer, adult	Patients without postoperative pneumonia	86/34	37	49		22	12		NA	ARMS-PCR	CCC
IL10 rs1800872 (-592A>C)						CC	CA	AA	CC	CA	AA			
Wattanathum	2005	Columbia (white)	Pneumonia complicated with sepsis, adult	Sepsis patients without pneumonia	392/158	212	137	43	89	61	8	**0.005**	Taqman	CCC
Endeman	2011	Netherlands	CAP, adult	PB	315/200	175	126	14	115	70	15	0.14	Taqman	CCB
Martinez-Ocana	2013	Mexico	Viral CAP, adult	PB	46/65	0	23	23	26	22	17	**0.024**	PCR-dot blot	CCC
Romanova	2013	Russia	Viral pneumonia, adult	NM	68/110	17	37	14	59	32	19	0.46	Allele-specific PCR	CCC
Wan	2013	China	Pneumonia in kidney transplant recipients, adult	Kidney transplant recipients without pneumonia	63/33	8	27	28	2	16	15	0.71	PCR-RFLP	CCC
TLR2 rs5743708 (2258 G>A, Arg753Gln)						GG	GA	AA	GG	GA	AA			
Moens	2007	Belgium	Pneumococcal disease, adult[d]	PB	170/72	165	5	0	67	5	0	0.85	PCR-RFLP	CCB
Yuan	2008	Australia,	Pneumococcal	NM	409/85	382	27	0	82	3	0	0.49	PCR-RFLP	CCC

		population data N/A	bacteraemia, pediatric[e]											
Carvalho	2009	Portugal	Viral pneumonia in CMV- infected patients after allogeneic hematopoietic stem cell transplantation, adult[f]	Non-infected patients after allogeneic hematopoietic stem cell transplantation	134/87	127	7	0	83	4	0	0.76	Bi-PASA	CCB
Endeman	2009	Netherlands	CAP, adult	PB	313/183	287	26	0	144	38	1	0.44	TaqMan	CCB
Esposito	2012	Italy	Viral pneumonia, pediatric	HB	164/18	161	3	0	17	1	0	0.91	TaqMan	CCA
Misch	2013	Netherlands	Legionnaires' disease, adult	PB	262/94	243	19	0	88	6	0	0.54	MassARRAY technique	CCB
Tellería-Orriols	2013	Spain	Pneumococcal disease, pediatric[g]	HB	66/92	49	15	2	37	46	9	0.53	PCR-RFLP	CCB
Dubinskaya	2014	Ukraine	Viral CAP, adult	Patients with viral infection without pneumonia	90/49	87	3	0	46	3	0	0.87	PCR	CCC
Schnetzke	2015	Germany	Pneumonia in AML patients after induction chemotherapy, adult	Non-infected AML patients after induction chemotherapy	104/51	102	2	0	43	8	0	0.92	Pyrosequencing	CCB
This study	2016	Russia	CAP, adult	PB	197/382	179	18	0	350	32	0	0.50	PCR-CTPP	CCA
This study	2016	Russia	HAP, adult	HB	210/347	193	17	0	329	18	0	0.54	PCR-CTPP	CCB
TLR4 rs4986790 (896A>G, Asp299Gly)						AA	AG	GG	AA	AG	GG			
Hawn	2005	Netherlands	Legionnaires' disease, adult	PB (at high risk for exposure to *Legionella*)	102/495	97	5	0	431	64	0	0.80	Sequenom MassARRAY	CCA
Moens	2007	Belgium	Pneumonia in invasive pneumococcal disease, adult[d]	PB	178/72	161	16	1	60	10	2	0.40	PCR-RFLP	CCB
Yuan	2008	Australia, population data N/A	Pneumococcal bacteraemia, pediatric[e]	NM	409/85	364	44	1	82	3	0	0.87	PCR-RFLP	CCC

Carvalho	2009	Portugal	Viral pneumonia in CMV- infected patients after allogeneic hematopoietic stem cell transplantation, adult[f]	Non-infected patients after allogeneic hematopoietic stem cell transplantation	134/87	116	18	0	81	6	0	0.41	Bi-PASA	CCB
Endeman	2009	Netherlands	CAP, adult	PB	313/200	280	32	1	171	27	2	0.93	TaqMan	CCB
Esposito	2012	Italy	Viral pneumonia, pediatric	HB	164/18	148	16	0	16	2	0	0.51	TaqMan	CCA
Tellería-Orriols	2013	Spain	Pneumococcal disease, pediatric[g]	HB	66/92	49	15	2	76	13	3	0.53	PCR-RFLP	CCB
Dubinskaya	2014	Ukraine	Viral CAP, adult	Patients with viral infection without pneumonia	90/49	87	3	0	42	7	0	0.87	PCR	CCC
Schnetzke	2015	Germany	Pneumonia in AML patients after induction chemotherapy, adult	Non-infected AML patients after induction chemotherapy	104/51	96	8	0	40	11	0	0.68	Pyrosequencing	CCB
TLR4 rs4986791 (196C>T, Thr399Ile)						CC	CT	TT	CC	CT	TT			
Hawn	2005	Netherlands	Legionnaires' disease, adult	PB (at high risk for exposure to *Legionella*)	102/495	97	5	0	431	64	0	0.80	Sequenom MassARRAY	CCA
Yuan	2008	Australia, population data N/A	Pneumococcal bacteraemia, pediatric[e]	NM	409/85	365	43	1	82	3	0	0.82	PCR-RFLP	CCC
Kumpf	2010	Greece	VAP[h]	NM	176/159	150	24	2	147	12	0	0.36	PCR-RFLP	CCA
Esposito	2012	Italy	Viral pneumonia, pediatric	HB	164/18	148	16	0	17	1	0	0.51	TaqMan	CCA
Schnetzke	2015	Germany	Pneumonia in AML patients after induction chemotherapy, adult	Non-infected AML patients after induction chemotherapy	104/51	96	8	0	40	11	0	0.68	Pyrosequencing	CCB
This study	2016	Russia	CAP, adult	PB	191/372	162	24	5	318	53	1	0.002	PCR-CTPP	CCA
This study	2016	Russia	HAP, adult	HB	209/343	167	41	1	300	43	0	0.36	PCR-CTPP	CCB
TLR9 rs187084 (-1486T>C)						TT	TC	CC	TT	TC	CC			

Carvalho	2009	Portugal	Viral pneumonia in CMV- infected patients after allogeneic hematopoietic stem cell transplantation, adult[f]	Non-infected patients after allogeneic hematopoietic stem cell transplantation	134/87	75	55	4	61	22	4	0.10	Bi-PASA	CCB
This study	2016	Russia	CAP, adult	PB	194/368	65	93	36	125	182	61	0.79	PCR-CTPP	CCA
This study	2016	Russia	HAP, adult	HB	207/340	57	106	44	104	171	65	0.68	PCR-CTPP	CCB
TLR9 rs5743836 (-1237T>C)						TT	TC	CC	TT	TC	CC			
Carvalho	2009	Portugal	Viral pneumonia in CMV- infected patients after allogeneic hematopoietic stem cell transplantation, adult[f]	Non-infected patients after allogeneic hematopoietic stem cell transplantation	134/87	103	29	2	57	26	4	0.98	Bi-PASA	CCB
This study	2016	Russia	CAP, adult	PB	196/382	164	30	2	302	75	5	0.64	PCR-CTPP	CCA
This study	2016	Russia	HAP, adult	HB	208/343	161	45	2	264	73	6	0.55	PCR-CTPP	CCB

[a]Pediatric and adult cases and controls have been age-matched unless otherwise specified.
[b]Data are graded by the Venice criteria for studies of genetic associations. Venice guidelines grade (1) the amount of evidence, (2) whether replication was performed and (3) protection from bias (Ioannidis et al. 2007).
[c]Sixty-one patients with community-acquired pneumonia, 5 patients with meningitis, and 3 patients with pneumonia and meningitis
[d]Pneumonia in invasive pneumococcal disease (n= 72; 72.7% of infected patients)
[e]Number of patients with pneumonia is not available
[f]CMV (cytomegalovirus) infection complicated with pneumonia (n=76; 87% of infected patients)
[g]Pneumonia and sepsis in invasive pneumococcal disease (n=92; 80.7% of infected patients)
[h]Age is not specified.

Abbreviations: AML, acute myeloid leukemia; ARMS-PCR, amplification refractory mutation system-polymerase chain reaction; Bi-PASA, bidirectional polymerase chain reaction amplification of specific alleles; HP, hospital based; HWE, Hardy-Weinberg equilibrium; PB, population based; NM, no mention; PCR-CTPP, polymerase chain reaction with confronting two-pair primers; PCR-RFLP, polymerase chain reaction–restriction fragment length polymorphism; SSP-PCR, single strand polymorphism polymerase chain reaction; VAP, ventilator associated pneumonia

Supplementary Table S4. Causative microorganisms in meta-analyzed studies

Pathogen	Sample (n)	%, whole sample	Disease	Reference	Genes under study
Streptococcus pneumonia	201	29.9%	CAP	Endeman, 2009, 2011	Interleukins, TLR genes
Streptococcus pneumonia	1227	24.6%	CAP	Martin-Loeches, 2012	Interleukins
Streptococcus pneumonia	390	36.6%	CAP	This study	Interleukins, TLR genes
Streptococcus pneumonia	355	5.4%	HAP	This study	Interleukins, TLR genes
Streptococcus pneumonia	69	100.0%	Pneumococcal disease	Schaaf, 2003	Interleukins
Streptococcus pneumonia	100	100.0%	Pneumococcal disease	Schaaf, 2005	Interleukins
Streptococcus pneumonia	99	100.0%	Pneumonia in invasive pneumococcal disease	Moens, 2007	TLR genes
Streptococcus pneumonia	85	100.0%	Pneumococcal bacteremia	Yuan, 2008	TLR genes
Streptococcus pneumonia	114	100.0%	Pneumonia and sepsis in invasive pneumococcal disease	Tellería-Orriols. 2014	TLR genes
Staphylococcus aureus	1227	1.7%	CAP	Martin-Loeches, 2012	Interleukins
Staphylococcus aureus	201	3.0%	CAP	Endeman, 2009	TLR genesgenes
Staphylococcus aureus	390	8.6%	CAP	This study	Interleukins, TLR genes
Staphylococcus aureus	355	13.5%	HAP	This study	Interleukins, TLR genes
Haemophilus influenza	201	7.0%	CAP	Endeman, 2009, 2011	Interleukins, TLR genes
Haemophilus influenza	1227	1.5%	CAP	Martin-Loeches, 2012	Interleukins
Haemophilus influenza	390	11.4%	CAP	This study	Interleukins, TLR genes
Haemophilus influenza	355	2.8%	HAP	This study	Interleukins, TLR genes
Legionella pneumophila	201	4.5%	CAP	Endeman, 2009, 2011	Interleukins, TLR genes
Legionella pneumophila	98	100.0%	Legionnaires’ disease	Misch, 2013	TLR genes
Pseudomonas aeruginosa	1227	2.1%	CAP	Martin-Loeches, 2012	Interleukins
Pseudomonas aeruginosa	355	15.8%	HAP	This study	Interleukins, TLR genes
Mycoplasma pneumonia	201	4.5%	CAP	Endeman, 2009, 2011	Interleukins, TLR genes
Klebsiella pneumoniae	201	2.0%	CAP	Endeman, 2009, 2011	TLR genesgenes
Klebsiella pneumoniae	355	13.8%	HAP	This study	Interleukins, TLR genes
Escherichia coli	390	4.8%	CAP	This study	Interleukins, TLR genes
Escherichia coli	355	2.0%	HAP	This study	Interleukins, TLR genes
Acinetobacter baumannii	355	4.2%	HAP	This study	Interleukins, TLR genes
Influenza A virus subtype H1N1	65	100.0%	CAP	Martinez-Ocaña, 2013	Interleukins
Influenza A virus subtype H1N1	111	100.0%	CAP	Romanova, 2013	Interleukins
Influenza A virus subtype H1N1/H3N2	49	100.0%	CAP	Dubinskaya, 2014	TLR genes
Influenza A virus subtype H1N1	18	100.0%	CAP	Esposito, 2012	TLR genes

CMV infection	87	100.0%	Pneumonia in CMV infected patients after allogeneic hematopoietic stem cell transplantation	Carvalho, 2009	TLR genes

Supplementary Table S5. Summary outcomes of sensitivity analysis for the association between *TLR2* rs5743708 (dominant model) and CAP/ Legionnaires' disease/ pneumococcal disease

Study omitted	Cases/ Controls (n)	I^2 (%)	Effect	OR [95% CI]	P
Dubinskaya	841/1172	70	Random	1.98 [1.05 – 3.73]	0.03
Endeman	707/949	63	Random	1.78 [0.88 – 3.57]	0.11
Esposito	872/1098	70	Random	1.92 [1.04 – 3.55]	0.04
Misch	796/1000	62	Random	2.28 [1.24 – 4.19]	0.008
Moens	818/1092	70	Random	1.91 [1.00 – 3.67]	0.05
Tellería-Orriols	798/1196	54	Random	1.64 [0.91 – 2.96]	0.10
This study (CAP)	508/1065	33	Fixed	2.64 [1.87 – 3.73]	<0.00001

Supplementary Table S6. Summary of genetic association studies of interleukin genes polymorphisms and *ex vivo* response to bacterial toxins

Reference	Year	Country, ethnicity	N Sample	Stimulation	Measure ment	SNPs	Genotyping	Results
					IL10			
Eskdale	1998	Denmark (Ca)	78 healthy	LPS-stimulated whole blood	ELISA	*IL10.R*, *IL10.G*[1]	PCR with a radioactive label	IL10.R2/G14 haplotype was associated with the highest IL10 secretion (vs. all others, *P* = 0.024); IL10.R3/G7 was associated with the lowest IL10 production (vs. all others, P = 0.006)
Crawley	1999	UK (Ca)	45 healthy	LPS-stimulated whole blood and PBMC	ELISA	-1082 G/A, -819 C/T, -592 C/A	SSOP	After LPS stimulation of the whole blood culture, ATA/ATA genotype was associated with the lowest IL10 production (P< 0.02)
Edwards-Smith	1999	Australia (Ca)	10 healthy	LPS-stimulated PBMC	ELISA	-1082 G/A, -819 C/T, -592C/A	PCR-RFLP	GCC haplotype was associated with higher IL10 production
Huizinga	2000	Netherlands (Ca)	158 healthy (61 families)	LPS-stimulated whole blood	ELISA, RT- PCR	-1082 G/A, -819 C/T, -592 C/A	PCR-dot blot	-1082 G was associated with lower IL10 production (P< 0.02) and mRNA expression (11 donors)
Koss	2000	UK (Ca)	52 healthy	LPS-stimulated whole blood	ELISA	-1082 G/A, -819 C/T, -592 C/A	PCR-RFLP	-1082A/A genotype and ATA haplotype were associated with lower IL10 production (P = 0.015 and P = 0.015, respectively)
Gibson	2001	USA (Ca and AA)	52 healthy Ca and 64 healthy AA	LPS-stimulated whole blood	ELISA, RT- PCR	Seven SNPs within the -1.3 kb – -4 kb promoter region of *IL10*	Sequencing	-3575T/-2849G/-2763C haplotype was associated with higher IL10 production (n=52) (P< 0.05) and mRNA production in CA
Reuss	2002	Germany (Ca)	57 DZ and 66 MZ adult twin pairs	LPS-stimulated whole blood	ELISA	-1082 G/A, -819 C/T, -592 C/A	PCR-RFLP	ns
Suárez	2003	Spain (Ca)	29 healthy	LPS-stimulated PBMC	ELISA	-1082 G/A, -819 C/T, -592C/A	RT-PCR	-1082 G/G genotype was associated with higher IL10 production (P = 0.008)
Temple	2003	Australia (Ca)	16 healthy	Stimulated PBMC (LPS, heat-killed *S. pneumoniae*)	RT- PCR	-1082 G/A, -819 C/T, -592 C/A	MassARRAY technique	LPS stimulated *IL10* levels, ns<br>After *S. pneumoniae* stimulation, ATA haplotype was associated with higher *IL10* mRNA levels than those with the GCC haplotype (P=0.024), or the ACC haplotype (P<0.0001)
George	2004	UK (Ca)	33 healthy	LPS-stimulated PBMC	ELISA, RT- PCR	-1082 G/A, -819 C/T,	PCR-SSP	-1082 G/G genotype was associated with higher mRNA expression (P< 0.05) and IL10 production (P< 0.02)

						-592 C/A, IL10.R, IL10.G[1]		
Mörmann	2004	Germany, mixed	107 healthy (A, Ca, As)	LPS-stimulated PBMC and LCL	ELISA	-6752 A/T, -6208 C/G, -3538 A/T, -2763 A/C, -1354 A/G, -1082 A/G, -819 C/T, -592 A/C, *IL10.R*, *IL10.G*[1]	Multiplex-PCR	–6752T/-6208C/-3538A haplotype and -1082 G allele were associated with higher IL10 production (P = 0.03 and P < 0.017, respectively)<br>IL10.G microsatellite with 22, 24 or 26 dinucleotide repeats (linked with the -1087G SNP) was associated with the highest levels of IL10 expression
Moscovis	2004	Australia and UK, mixed	69 smokers and 43 non-smokers	LPS- and TSST-stimulated whole blood	ELISA	-1082 G/A	RT-PCR	ns
Schippers	2005	Netherlands (Ca)	125 patients before CTS with CPB	LPS-stimulated whole blood (three LPS concentrations)	ELISA	-3575 T/A, -2849 G/A, -2763 C/A, -1330 A/G, -1082G/A, -819C/T, -592C/A	PCR-dot blot	-1330G/-1082A/-819T/-592A haplotype was associated with lower IL10 levels under 10 ng/mL LPS (P = 0.041) and 100ng/mL LPS (P=0.006)<br>1000 ng/mL LPS, ns
Yılmaz	2005	Turkey (Ca)	Healthy (PCR, n=152, ELISA, NM)	Stimulated PBMC (PPD, LPS, SAC)	ELISA	-3575 T/A, -2763 C/A, -1082 G/A, -819 C/T, -592 C/A	PCR-RFLP and PCR-SSP	After stimulation with PPD or SAC, -1082 A allele was associated with lower levels of IL10 (P = 0.006 and P = 0.04, respectively)<br>After LPS, ns
Allen	2006	UK (Ca)	36 pediatric patients before and after CS with CPB	LPS-stimulated whole blood	ELISA	-1082 G/A, -819 C/T, -592 C/A	PCR-RFLP	ns
Stanilova	2006	Bulgaria (Ca)	28 healthy and 24 sepsis patients	LPS-stimulated PBMC	ELISA	-1082 G/A	ARMS-PCR	AG/GG genotype was associated with higher levels of IL10 in patients and in controls
Aborsangay	2007	Canada,	53 healthy	β-gal-stimulated	ELISA	-1082 G/A, -	PCR with	-819/-592 C/C alleles were associated with lower IL10

a		mixed	(29 Ca and 24 NA)	PBMC		819 C/T, -592 C/A	sequence-specific primers	production in CA (P<0 .005) and FN (P < 0.005) -1082 G allele was associated with lower β-gal-stimulated IL10 production in CA (P < 0.005)
Steinke	2007	USA (NM)	17 healthy	LPS-stimulated monocytes/T cells/B cells	ELISA, RT- PCR	-592 C/A	EMSA	C/C genotype was associated with higher IL10 production in monocytes and B-cells.
Rosado	2008	Spain (Ca, mixed)	17 healthy	LPS-stimulated monocytes	ELISA	*IL10.R*, *IL10.G*,[1] -1082 G/A, -819 C/T, -592 C/A	PCR-RFLP	ns
Salhi	2008	Brazil (NM)	28 healthy	Stimulated PBMC (LPS, HA)	ELISA	-819 C/T	PCR-RFLP	C/C genotype was associated with higher IL10 production after LPS stimulation (P = 0.03) and hyaluronic acid stimulation (P = 0.05)
Zeng	2009	China (As)	308 major trauma patients	LPS-stimulated whole blood	ELISA	-1082 G/A, -819 C/T, -592 C/A	PCR-RFLP	-1082A and -592A alleles were significantly associated with lower LPS induced IL-10 production in an allele-dose dependent fashion (P = 0.003 and P = 0.037, respectively)
Pereira	2009	Brazil (NM)	Healthy (PCR, n=380, ELISA, NM)	Stimulated PBMC (LPS, *Mycobacterium leprae* antigens)	ELISA	-3575 T/A, -2849 G/A, -2763 C/A, -819 C/T	PCR-RFLP	LPS, ns -819T carriers produced lower levels of IL-10 after *Mycobacterium leprae* stimulation (P <0.05)
Yan	2009	China (As)	26 healthy	LPS-stimulated PBMC	ELISA, RT- PCR	-1082 G/A, -819 C/T, -592 C/A	PCR-RFLP	-592 C/C-C/A genotype was associated with higher mRNA expression (P = 0.001)
Bos	2010	Netherlands (Ca)	563 participants over 85 years	LPS-stimulated whole-blood sample	ELISA	The genome-wide linkage analysis		No evidence for linkage was observed at the locus encoding IL10 (1q32.1)
Huebinger	2010	USA, mixed	31 healthy	LPS-stimulated whole blood	ELISA	-819 C/T, -592 C/A	RT-PCR (TaqMan)	ns
Remmers	2010	Turkey (NM)	38 healthy	LPS-stimulated PBMC	ELISA	GWAS		*IL10* rs1518111 G/G genotype was associated with higher IL10 production (vs. A/A genotype, P = 0.0001)
Boonnak	2011	USA (NM)	9 healthy	LPS-stimulated monocytes	ELISA, RT- PCR	-1082 G/A, -819 C/T, -592 C/A	PCR-RFLP	High-level (GCC), intermediate (ACC), and low-level (ATA) haplotypes were associated with IL10 protein and corresponding RNA levels

Boef	2012	Netherlands (A)	111 subjects in an environment with high infectious pressure (Ghana)	Repeated measurements (2006 and 2008 years) in stimulated whole blood (LPS + zymosan)	ELISA	20 SNPs in the *IL10* gene region	NM	-819T/ -592A/504T/1548T haplotype was associated with lower IL10 production ($P< 0.05$)
Nur	2012	Turkey (Ca)	32 healthy (≤ 6 y)	LPS-stimulated PBMC	ELISA	-1082 G/A	ARMS-PCR	ns
Larsen	2013	Denmark (Ca)	130 (discovery set)	LPS-stimulated whole blood	Fluorokine® MAP-assay	GWAS		ns
Stappers	2014	Netherlands (Ca)	66 healthy	Stimulated PBMC (heat-killed *Staphylococcus aureus* and *E. coli*)	ELISA	-819T>C	RT-PCR (TaqMan)	ns
				IL6				
Kilpinen	2001	Finland (Ca)	50 healthy newborns, healthy adults (PCR, n=450, ELISA, NM)	LPS-stimulation in cord blood	ELISA	-174G>C	PCR-RFLP	Higher IL6 production in the carriers of C/C genotypes than in the individuals with the G/C-G/G genotypes; in adults, ns
Heesen	2002	Germany (Ca)	89 trauma patients	LPS-stimulated whole blood	ELISA	-174G>C	RT-PCR	ns
Rivera-Chavez	2003	USA (Ca)	49 healthy	LPS-stimulated whole blood	EMSA	-597G>A, -572 G>C, -174G>C	Pyrosequencing, EMSA	Haplotype -597G/-174G was associated with the highest level of IL6 production; haplotype -597A/-174C was associated with the lowest level of IL6 secretion
Kim	2005	Korea (As)	110 postmenopausal women	LPS-stimulated whole blood	ELISA	-572 G>C	PCR-RFLP	ns
Kiszel	2007	Hungary (Ca)	33 umbilical cords from healthy pregnancies	Stimulated HUVEC (IL-1β, LPS)	ELISA	-174G>C	PCR-RFLP	ns
Gu	2010	China (As)	308 major trauma	LPS-stimulated whole	ELISA	-572 G>C	PCR-RFLP	ns

			patients	blood				
Patel	2010	USA (NM)	31 healthy	Stimulated PBMC (RSV, LPS)	NM	-174G>C	PCR-RFLP	Higher IL6 production in the carriers of G/C- C/C genotypes than in wild-type (G/G) participants
Noss	2015	USA (NM)	10 healthy	Stimulated skin, synovial fibroblasts, $CD14^+$ monocytes (TNF-α, IL-1β, LPS)	RT- PCR	-174G>C	PCR-RFLP	*IL6* expression was significantly associated with the minor allele (CC) genotype in fibroblasts but not $CD14^+$ monocytes
				IL8				
Hull	2000	UK (Ca)	50 healthy	LPS-stimulated whole blood	ELISA	-251A>T	Sequencing and ARMS-PCR	ns
Gu	2010	China (As)	308 major trauma patients	LPS-stimulated whole blood	ELISA	-251A>T	PCR-RFLP	IL8 expression was higher in the carriers of T allele (P<0.001)
				IL1B				
Pociot	1992	Denmark (Ca)	29 healthy	Stimulated PBMC (LPS, PPD, PHA)	ELISA	+3953 C>T	PCR-RFLP	IL1B LPS-stimulated expression was higher in the carriers of T allele;<br>PPD and PHA, ns
Vamvakopoulos	2002	UK (Ca)	55 healthy	LPS-stimulated PBMC	ELISA	-31C>T, +3953 C>T	ARMS-PCR	ns
Hernandez-	2003	Mexico (NM)	22 amniochorions (healthy)	LPS- stimulated membranes	ELISA	+3953 C>T	PCR-RFLP	Secretion of IL1B by membranes carrying a T allele was higher than for those with C allele; significant differences were found at 5.0, 10.0 and 50.0 ng/ml LPS ($P < 0.05$).
Schrijver	2003	Netherlands (Ca)	18 healthy	LPS + ATP stimulated whole blood	NM	+3953 C>T	PCR-RFLP	ns
Hall	2004	Netherlands (Ca)	31 healthy (30 Ca + 1A)	LPS-stimulated whole blood	ELISA	−511C/T, −31C/T, +3953C/T	PCR-RFLP	Haplotype -511T/-31C was associated with an increase in LPS-induced IL1B protein secretion (P = 0.0084)
Awomoyi	2005	Gambia (A)	250 healthy	Stimulated whole blood (LPS alone or LPS + IFN-γ)	ELISA	-511 C>T, +3953 C>T	PCR-RFLP	Secretion of IL1B stimulated by LPS + IFN-γ was higher in the carriers of -511T/C-C/C genotypes and 3953 T/T-T/C genotypes

Iacoviello	2005	Italy (Ca)	145 healthy	LPS-stimulated PBMC	ELISA	−511C/T, −31C/T, +3953C/T	PCR-RFLP	-511C allele was associated with higher IL1B concentrations in an allele-dose dependent fashion
Chen	2006	USA (mixed)	25 healthy	LPS-stimulated transfected THP-1 cells	Transient transfection reporter gene assay and EMSA	-3737 C>T, -1464 G>C, -511 C>T, -31C>T	Sequencing	-1464C and -31C alleles were associated with decreased transcriptional activity (TA), while -511T allele was associated with increased TA. Nuclear protein binding was lower for -3737T allele, higher for -1464C allele and varied for -31C allele in relation to different complexes
Wen	2006	China (As)	105 healthy	LPS-stimulated whole blood	ELISA	-1470G>C, -511 C>T, -31C>T	PCR-RFLP	Haplotype -1470G/-511C/-31T was associated with higher IL1B concentration than haplotype -1470C/-511T/-31C
Latella	2009	Italy (Ca)	64 healthy	LPS-stimulated PBMC	ELISA	-3737 C>T, -1464 G>C, -511 C>T,	RT-PCR (TaqMan)	Haplotypes -3737C/-1464C/-511T and -3737C/-1464G/-511T were associated with lower IL1B concentations
Gu	2010	China (As)	308 major trauma patients	LPS-stimulated whole blood	ELISA	-1470G>C, -511 C>T, -31C>T	PCR-RFLP	IL1B expression was lower in the carriers of-1470 CC genotype (P=0.01); IL1B expression was higher in the carriers of-31TT genotype (P<0.010)
IL4								
Gu	2010	China (As)	308 major trauma patients	LPS-stimulated whole blood	ELISA	-589T>C	PCR-RFLP	IL4 expression was higher in the carriers of-589C allele (P<0.001)

Abbreviations: A, Africans; AA, African Americans; ARMS-PCR, amplification refractory mutation system; As, Asians; ATP, Adenosine triphosphate; Ca, Caucasians; ConA, concanavalin A; CPB, cardiopulmonary bypass; CS, cardiac surgery; CTS, cardiothoracic surgery; DZ, dizygotic; EMSA, electrophoretic mobility shift assay; GWAS, genome-wide association study; HA, hyaluronic acid; HUVEC, human umbilical cord vein endothelial cells; LCL, lymphoblastoid cell line; LPS, lipopolysaccharide; MZ, monozygotic; NA, native Americans; NM, no mention; PHA, phytohemagglutinin; PPD, purified protein derivative (from *E. coli*); RSV, respiratory syncytial virus; RT, real-time; SAC, *Staphylococcus aureus* Cowan strain I; β-gal, β-galactosidase; TSST, toxic shock syndrome toxin-1, secreted by *Staphylococcus aureus*; PBL, peripheral blood lymphocytes; PBMC, peripheral blood mononuclear cells; PCR-SSP, polymerase chain reaction-sequence-specific primer; SSOP, sequence-specific oligonucleotide probing; y; years

IL10 SNPs: –6752 A/T (rs6676671), –6208 C/G (rs10494879), - 3575 A/T (rs1800890), -3538 A/T (rs1800890), - 2763 C/A (rs6693899),- 2849 G/A (rs6703630), -1330 A/G (rs1800893), -1082 A/G (rs1800896), - 819 C/T (rs1800871), -592 A/C (rs1800872), 504 G/T (rs3024490), 1548 C/T (rs1554286); *IL6* SNPs: -597G>A (rs1800797), -572 G>C (rs1800796), -174G>C (rs1800795); *IL8* SNP: -251A>T (rs4073); *IL-1b* SNPs: -3737 C>T (rs4848306); -1470G>C/-1464G>C (rs1143623); -511 C>T (rs16944); -31C>T (rs1143627); 3953C>T (rs1143634); *IL4* SNP: -589C>T (rs2243250)

[1]CA-repeat microsatellites, IL-10.R (−4 kb) and IL-10.G (−1.1 kb)

Supplementary Table S7. Interleukin and toll-like receptor genes mRNA expression data for the available probes by the genotypes of SNPs representing corresponding haplotypes[a]

Gene, SNP	Genotypes	Counts	Mean	SD	P-value
IL1B rs1143633	T/T	9	7.4	0.9109	0.07589
	T/C	28	7.397	0.7164	
	C/C	21	6.992	0.6146	
IL6 **rs1800795**	G/G	14	6.143	0.1828	0.02085
	G/C	28	6.338	0.37	
	C/C	18	6.417	0.3662	
IL6 rs2069832	G/G	14	6.143	0.1828	0.02085
	G/A	28	6.346	0.37	
	A/A	17	6.433	0.3662	
IL6 rs1474347	A/A	14	6.143	0.1828	0.02055
	A/C	29	6.332	0.3712	
	C/C	17	6.433	0.3662	
IL6 rs1554606	G/G	13	6.148	0.1891	0.03606
	G/T	29	6.329	0.3727	
	T/T	18	6.417	0.3619	
IL4 rs2227284	T/T	3	6.75	0.2789	0.05973
	T/G	26	6.55	0.1599	
	G/G	30	6.514	0.1617	
IL4 rs2227282	C/C	3	6.75	0.2789	0.05973
	C/G	26	6.55	0.1599	
	G/G	30	6.514	0.1617	
IL4 rs2243270	G/G	2	6.827	0.3463	0.08717
	G/A	16	6.543	0.1852	
	A/A	42	6.523	0.1503	
IL13 rs1295686	T/T	3	6.762	0.5989	0.06592
	T/C	22	6.235	0.2745	
	C/C	35	6.209	0.3279	
IL13 **rs20541**	A/A	3	6.762	0.5989	0.06592
	A/G	22	6.235	0.2745	
	G/G	35	6.209	0.3279	
IL10 rs3024496	A/A	12	7.767	0.354	0.03898
	A/G	29	7.735	0.3835	
	G/G	17	7.508	0.2707	
IL10 rs1878672	G/G	13	7.742	0.3504	0.04519
	G/C	31	7.742	0.3723	
	C/C	16	7.492	0.2706	
IL10 rs1554286	A/A	1	7.998	0	0.05955
	A/G	18	7.789	0.3878	
	G/G	40	7.621	0.337	
IL10 rs1518111	T/T	2	7.793	0.2896	0.09132
	T/C	20	7.78	0.377	
	C/C	36	7.61	0.3448	
IL10 rs2222202	G/G	13	7.742	0.3504	0.07856
	G/A	30	7.745	0.3784	
	A/A	14	7.503	0.2861	
IL10 rs1800872	T/T	2	8.306	0.9292	0.09034
	T/G	19	8.202	0.6897	
	G/G	36	7.94	0.4896	
IL10 **rs1800896**	T/T	13	7.742	0.3504	0.05492
	T/C	30	7.741	0.3786	
	C/C	17	7.508	0.2707	
IL10 rs1800893	C/C	13	7.742	0.3504	0.05557
	C/T	28	7.752	0.3828	
	T/T	17	7.508	0.2707	
TLR4 rs5030717	G/G	1	6.243	0	0.06445

	G/A	14	6.212	0.07696
	A/A	45	6.271	0.09725

[a]Genotyping data and mRNA expression levels by genotypes were obtained from the HapMap phase II release 23 data from EBV-transformed lymphoblastoid cell lines from 60 CEU parents.
Data are given for the results with significance level P<0.10 under additive model. SNPs considered in this study are in bold. Probes: *IL1B*, GI_27894305-S; *IL6*, GI_10834983-S; *IL4*, GI_27477091-A; *IL13*, GI_26787977-S; *IL10*, GI_24430213-S and GI_24430216-S; *TLR4*, GI_19924152-A

Supplementary Table S9. Functional characteristics of the SNPs in the genes under study

Gene	SNP	Chromosome: position (GRCh38.p2)	RegPotential[a]	Regulome DB Score[b]	The NHGRI GWAS Catalog associations[c]
L10	rs3024500	1:206767486	0.193179	6	No
IL10	rs3024498	1:206768184	0.157078	4	No
IL10	rs3024496	1:206768519	0.151515	5	No
IL10	rs3024495	1:206769068	N/A	4	No
IL10	rs3024509	1:206769952	0.060395	3a	No
IL10	rs1878672	1:206770368	0.0	4	No
IL10	rs3024493	1:206770623	0.041557	2b	Yes[d]
IL10	rs3024492	1:206770767	0.057937	4	No
IL10	rs1554286	1:206770888	0.155715	1f	No
IL10	rs1518111	1:206771300	0.241228	3a	Yes[e]
IL10	rs1518110	1:206771516	0.0	3a	No
IL10	rs3021094	1:206771607	0.255299	4	No
IL10	rs3024490	1:206771966	0.0	6	No
IL10	rs2222202	1:206772036	0.0	5	No
IL10	rs1800872	1:206773062	0.078963	5	No
IL10	rs1800871	1:206773289	0.0	3a	Yes[f]
IL10	rs1800896	1:206773552	0.0	6	No
IL10	rs1800893	1:206773822	0.0	5	No
IL1B	rs1071676	2:112829856	0.026017	6	No
IL1B	rs1143643	2:112830725	0.0	6	No
IL1B	rs1143639	2:112831216	0.074026	5	No
IL1B	rs1143637	2:112831756	0.055559	3a	No
IL1B	rs1143634	2:112832813	0.183937	5	No
IL1B	rs1143633	2:112832890	0.0	4	No
IL1B	rs3136558	2:112833698	N/A	5	No
IL1B	rs3136557	2:112833765	N/A	5	No
IL1B	rs3917356	2:112834786	0.104633	2b	No
IL1B	rs1143629	2:112835941	0.095585	4	No
IL1B	rs1143627	2:112836810	0.138647	1b	No
IL1B	rs16944	2:112837290	0.202817	1f	No
IL1B	rs1143625	2:112837782	0.0	5	No
IL1B	rs1143623	2:112838252	0.08717	7	No
TLR9	rs5743846	3:52221672	0.378665	5	No
TLR9	rs352140	3:52222681	0.26582	5	No
TLR9	rs352139	3:52224356	0.196359	5	No
TLR9	rs5743836	3:52226766	0.0	5	No
TLR9	rs187084	3:52227015	0.0	1f	No
TLR2	rs4696480	4:153685974	N/A	5	No
TLR2	rs5743687	4:153686215	N/A	6	No
TLR2	rs1898830	4:153687301	N/A	7	No
TLR2	rs4696483	4:153698103	N/A	5	No

TLR2	rs11938228	4:153700794	N/A	7	No
TLR2	rs3804099	4:153703504	0.152475	7	No
TLR2	rs3804100	4:153704257	0.10659	7	No
TLR2	rs5743704	4:153704799	0.353217	5	No
TLR2	rs5743708	4:153705165	0.323433	7	No
TLR2	rs7656411	4:153706503	0.0	6	No
IL8	rs4073	4:73740307	0.0	2b	No
IL8	rs2227307	4:73740952	0.0	4	No
IL8	rs2227306	4:73741338	0.0	3a	No
IL8	rs2227543	4:73742193	0.0	1f	No
IL8	rs1126647	4:73743328	0.0	6	No
IL13	rs1881457	5:132656717	0.0	3a	No
IL13	rs1800925	5:132657117	0.150674	2b	No
IL13	rs2066960	5:132658743	0.0	4	No
IL13	rs1295686	5:132660151	0.108821	3a	Yes[g]
IL13	rs20541	5:132660272	0.021634	3a	Yes[h]
IL13	rs1295685	5:132660753	0.043141	4	No
IL13	rs848	5:132660808	0.082331	4	No
IL13	rs847	5:132660977	0.118299	6	No
IL4	rs2243238	5:132671728	N/A	7	No
IL4	rs2243243	5:132672565	N/A	5	No
IL4	rs2243307	5:132672580	N/A	5	No
IL4	rs2243248	5:132672952	0.068851	5	No
IL4	rs2243250	5:132673462	0.0	5	No
IL4	rs2070874	5:132674018	0.097197	2b	No
IL4	rs734244	5:132675034	0.190309	4	No
IL4	rs2227284	5:132677033	0.0	2b	No
IL4	rs2227282	5:132677487	0.0	3a	No
IL4	rs2243263	5:132677607	0.0	2b	No
IL4	rs2243266	5:132678097	0.0	4	No
IL4	rs2243267	5:132678194	0.0	3a	No
IL4	rs2243268	5:132678271	0.0	4	No
IL4	rs2243270	5:132678417	0.0	7	No
IL4	rs2243274	5:132679140	0.060255	5	No
IL4	rs2243281	5:132680703	N/A	5	No
IL4	rs2243282	5:132680862	0.0	3a	No
IL4	rs2243284	5:132681300	0.0	3a	No
IL4	rs2243285	5:132681301	0.0	3a	No
IL4	rs2243288	5:132682252	0.182399	3a	No
IL4	rs2243289	5:132682440	0.0	5	No
IL4	rs2243290	5:132682477	0.0	6	No
IL6	rs2069827	7:22725837	0.019139	4	No
IL6	rs1800797	7:22726602	0.226647	4	No
IL6	rs1800795	7:22727026	0.136017	4	No
IL6	rs2069832	7:22727814	0.182766	1d	No
IL6	rs2069833	7:22728045	0.0	5	No

IL6	rs1474348	7:22728289	0.10498	5	No
IL6	rs2069837	7:22728408	0.031117	2b	No
IL6	rs1474347	7:22728505	0.0	1f	No
IL6	rs2069840	7:22728953	0.0	5	No
IL6	rs1554606	7:22729088	0.139902	5	No
IL6	rs2069845	7:22730530	0.0	5	No
IL6	rs2069848	7:22731118	0.0	3a	No
IL6	rs2069861	7:22732035	0.0	5	No
TLR4	rs1927914	9:117702447	0.0	6	No
TLR4	rs10759932	9:117702866	N/A	7	No
TLR4	rs1927911	9:117707776	0.0	5	No
TLR4	rs11536878	9:117709275	0.0	6	No
TLR4	rs12377632	9:117710452	0.0	6	No
TLR4	rs1927907	9:117710486	0.0	7	No
TLR4	rs2770146	9:117711060	0.0	7	No
TLR4	rs5030717	9:117711556	0.0	7	Yes[i]
TLR4	rs2149356	9:117711921	0.0	6	No
TLR4	rs5030728	9:117712004	0.010763	7	No
TLR4	rs4986790	9:117713024	0.04955	6	No
TLR4	rs4986791	9:117713324	0.101936	7	No
TLR4	rs5030719	9:117713658	0.195137	7	No
TLR4	rs11536889	9:117715853	0.0	4	No
TLR4	rs7873784	9:117716658	N/A	7	No
TLR4	rs11536891	9:117717059	0.0	5	No
TLR4	rs11536896	9:117717456	N/A	6	No
TLR4	rs11536897	9:117717732	0.0	5	No
TLR4	rs1927906	9:117717837	0.0	7	No
TLR4	rs11536898	9:117717932	0.0	5	No

SNPs under study are signed in red.

[a] In this series, RegPotential from the SNPinfo resource (https://snpinfo.niehs.nih.gov/snpinfo/snpfunc.htm) varies in the range of 0.0 to the maximum value of 0.379.

[b]Regulome DB Score from RegulomeDB web server (http://regulomedb.org/) RegulomeDB presents a scoring system, with categories ranging from 1 to 7, where category 7 variants lack evidence of regulatory function, while category 1 variants are assumed to affect binding and expression of a gene target. Categories 1-3 are further divided into subcategories. A variant scored as 1a has the highest confidence on functionality.

[c]Associations from the NHGRI GWAS Catalog (http://www.ebi.ac.uk/gwas/):

[d]Ulcerative colitis; [e]Behcet's disease; [f]Behcet's disease; [g]Atopic dermatitis, Asthma (childhood onset), [h]Asthma; Psoriasis, Self-reported allergy, Hodgkin's lymphoma, IgE levels; [i]Plasma omega-6 polyunsaturated fatty acid levels (dihomo-gamma-linolenic acid)

N/A, not available

Supplementary Figure S1. Flow diagram of study selection. Literature search was performed in compliance with MOOSE (Meta-analysis Of Observational Studies in Epidemiology) (Stroup et al. 2000) and PRISMA guidelines (Preferred Reporting Items for Systematic Reviews and Meta-Analysis) (Moher et al. 2009).

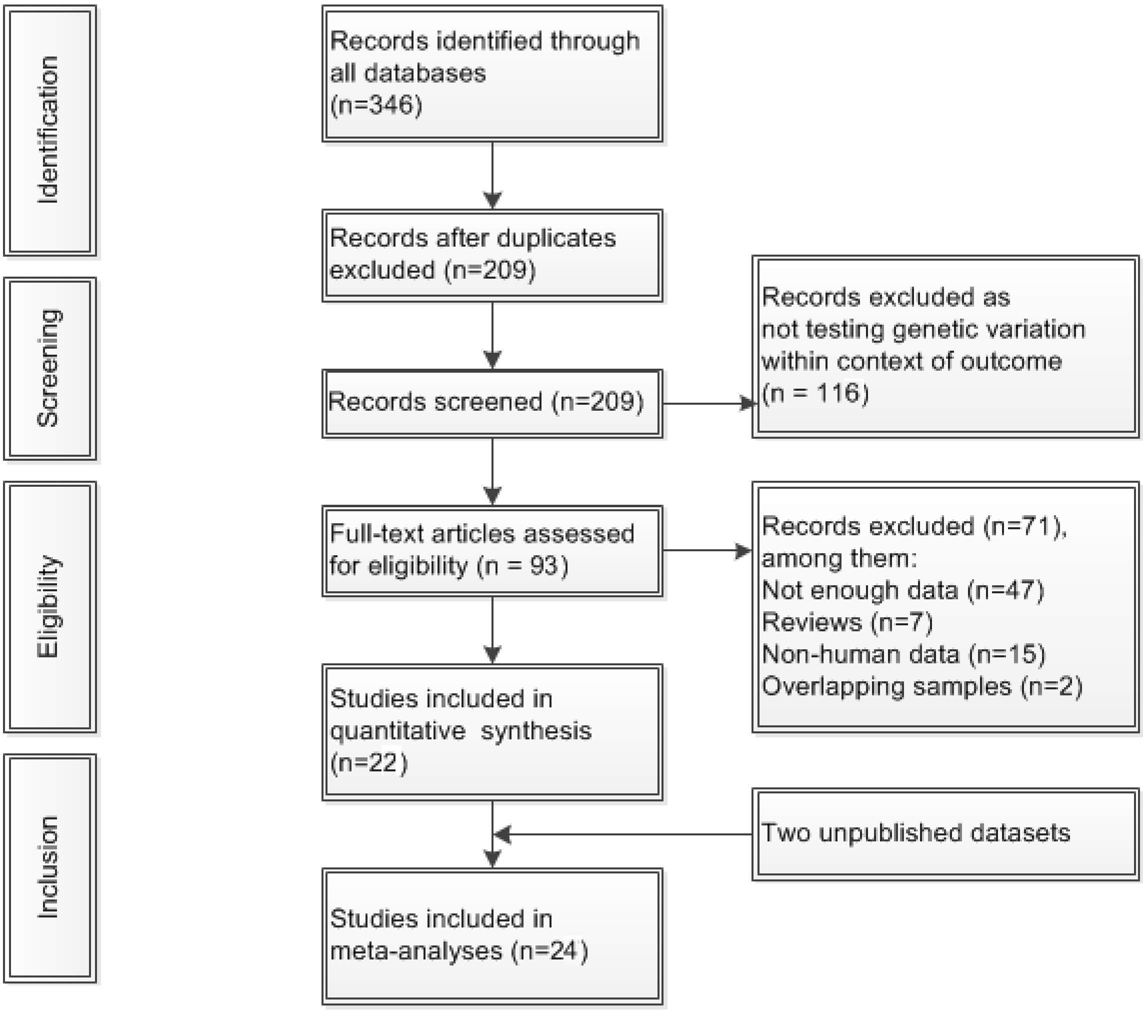

Supplementary Figure S2. Flow diagram of literature search for the studies of induced allele-specific cytokine expression profile

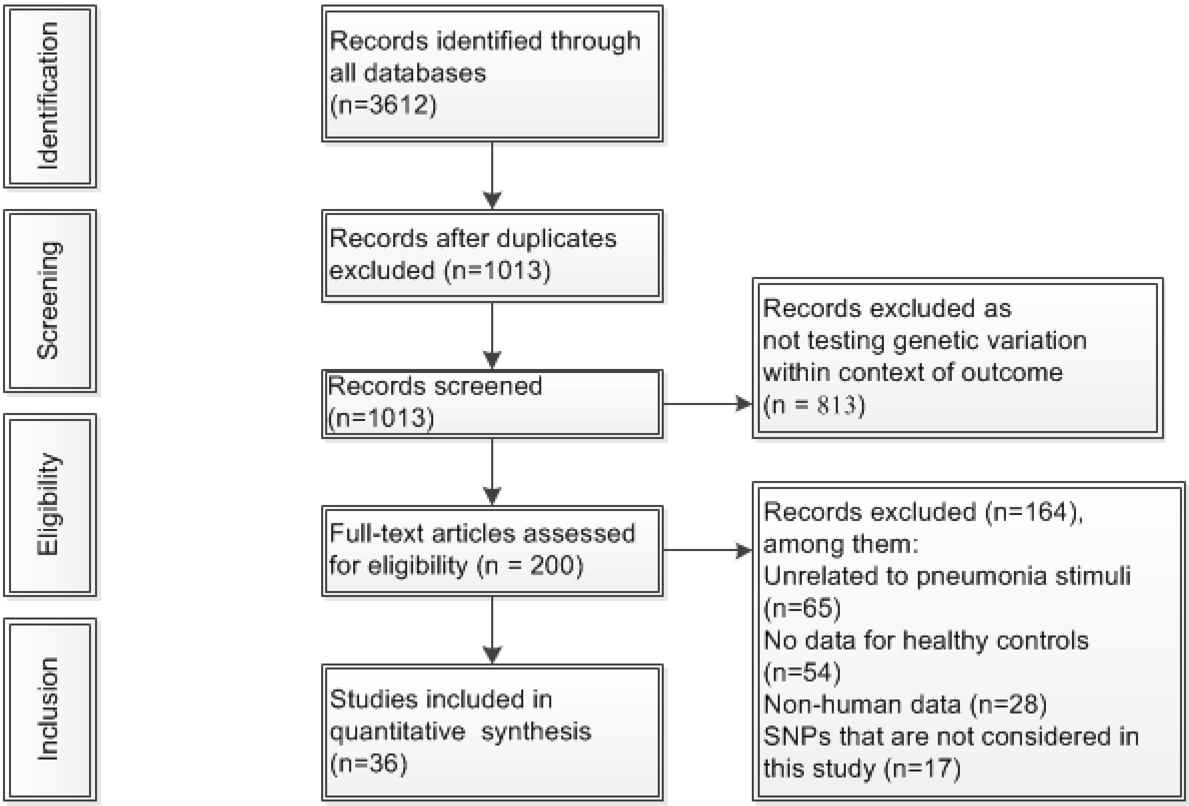

Supplementary Figure S3. Linkage disequilibrium plots of the *IL1B, IL8, IL4, IL13, TLR4* and *TLR9* gene regions generated with the LD tag SNP selection tool within SNPinfo resource. Pair-wise LD values are indicated by different color, which changes from red to white as the D' value decreases. SNP name is colored with genomic context: black, intron region; red: coding region; blue, UTR (untranslated) region, green, non-genic region. Minor allele frequency for each SNP in the European population is denoted by the height of green bar.

Functional annotations from the SNP info web server are shown with red arrows: one arrow, regulatory potential is in the range of 0.10– 0.20; two arrows, regulatory potential is in the range of 0.20– 0.30; three arrows, regulatory potential is higher than 0.30.

Functional annotations from the RegulomeDB info web server are represented with dark blue arrows: one arrow, score 2a–2f; two arrows, score 1a–1f. Scores 3–7 are not provided. SNPs with category 7 score lack evidence of regulatory function, while category 1 variants are assumed to affect binding and expression of a gene target. Within subcategories a–f, variant scored as 1a has the highest confidence on functionality.

Functional annotations from the NHGRI GWAS Catalog are marked by green arrows. The number of arrows corresponds to the number of associations in the Catalog.

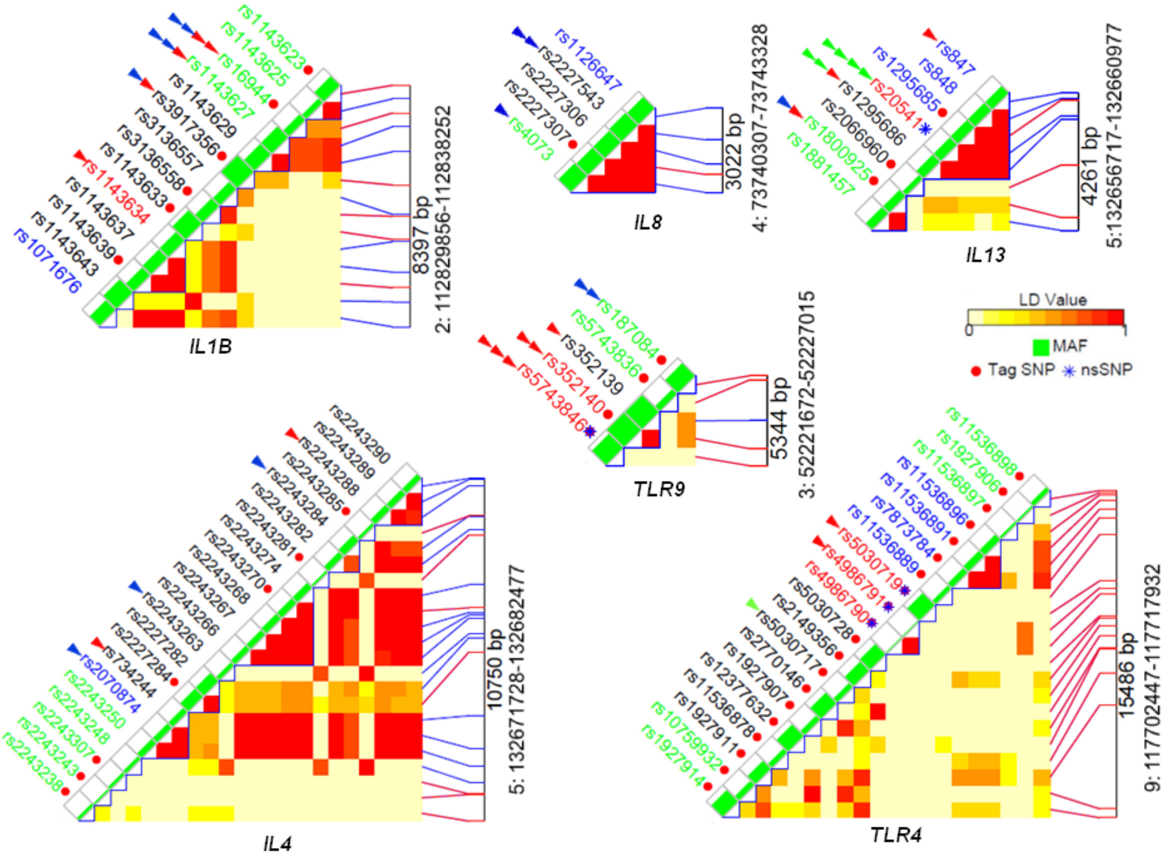

Supplementary References

1. Aborsangaya, K. B. et al. Impact of aboriginal ethnicity on HCV core-induced IL-10 synthesis: interaction with IL-10 gene polymorphisms. *Hepatology.* **45,** 623–630.
2. Allen, M. L. et al. Interleukin-10 and its role in clinical immunoparalysis following pediatric cardiac surgery. *Crit Care Med.* **34,** 2658–2665 (2006).
3. Awomoyi, A. A. et al. Polymorphism in IL1B: IL1B-511 association with tuberculosis and decreased lipopolysaccharide-induced IL-1beta in IFN-gamma primed ex-vivo whole blood assay. *J Endotoxin Res.* **11,** 281–286 (2005).
4. Boef, A. G. et al. The influence of genetic variation on innate immune activation in an environment with high infectious pressure. *Genes Immun.* **13,** 103–108 (2012).
5. Boonnak, K., Dambach, K. M., Donofrio, G. C., Tassaneetrithep, B. & Marovich, M. A. Cell type specificity and host genetic polymorphisms influence antibody-dependent enhancement of dengue virus infection. *J Virol.* **85,** 1671–1683 (2011).
6. Bos, S. D. et al. A genome-wide linkage scan reveals CD53 as an important regulator of innate TNF-alpha levels. *Eur J Hum Genet*. **18,** 953–959 (2010).
7. Carvalho, A. et al. Polymorphisms in Toll-like receptor genes and susceptibility to infections in allogeneic stem cell transplantation. *Exp. Hematol.* **37,** 1022–1029 (2009).
8. Chen, H. et al. Single nucleotide polymorphisms in the human interleukin-1B gene affect transcription according to haplotype context. Hum Mol Genet. **15,** 519–529 (2006).
9. Crawley, E. et al. Polymorphic haplotypes of the interleukin-10 5' flanking region determine variable interleukin-10 transcription and are associated with particular phenotypes of juvenile rheumatoid arthritis. *Arthritis Rheum.* **42,** 1101–1108 (1999).
10. Dubinskaya, G., Pryimenko, N.., Kaidashev, L., Pokhylko V. and Chub K. The role of TLR-2, TLR-3, TLR-4 genes polymorphism of grippe. *Georgian Med News.* **232–233,** 51–55 (2014).
11. Edwards-Smith, C. J. et al. Interleukin-10 promoter polymorphism predicts initial response of chronic hepatitis C to interferon alfa. *Hepatology.* **30,** 526–530 (1999).
12. Endeman, H. et al. Systemic cytokine response in patients with community-acquired pneumonia. *Eur Respir J.* **37,** 1431–1438. (2011).
13. Endeman H. *Clinical characteristics and innate immunity in patients with community-acquired pneumonia.* (2009) Utrecht University Repository (Dissertation) Supervisor(s): Biesma, D.H.; Bosch, J.M.M. van den; Grutters, J.C.; Voorn, G.P.
14. Eskdale, J. et al. Interleukin 10 secretion in relation to IL-10 locus haplotypes. *Proc Natl Acad Sci U S A.* **95,** 9465–9470 (1998).
15. Esposito, S. et al. Toll-like receptor 3 gene polymorphisms and severity of pandemic A/H1N1/2009 influenza in otherwise healthy children. *Virol. J.* **9,** 270 (2012).
16. George, S. et al. Renovascular disease is associated with low producer genotypes of the anti-inflammatory cytokine interleukin-10. *Tissue Antigens*. **63,** 470–475 (2004).
17. Georgitsi, M. D. et al. Individualized significance of the -251 A/T single nucleotide polymorphism of interleukin-8 in severe infections. *Eur J Clin Microbiol Infect Dis.* **35,** 563–570 (2016).
18. Gibson, A. W. et al. Novel single nucleotide polymorphisms in the distal IL-10 promoter affect IL-10 production and enhance the risk of systemic lupus erythematosus. *J Immunol.* **166,** 3915–3922 (2001).
19. Gu, W. et al. Clinical relevance of 13 cytokine gene polymorphisms in Chinese major trauma patients. *Intensive Care Med.* **36,** 1261–1265 (2010).
20. Hall, S. K. et al. Correlation of polymorphic variation in the promoter region of the interleukin-1 beta gene with secretion of interleukin-1 beta protein. *Arthritis Rheum.* **50,** 1976–1983 (2004).

21. Hawn, T. R. et al. Toll-like receptor 4 polymorphisms are associated with resistance to Legionnaires' disease. *Proc Natl Acad Sci U S A.* **102,** 2487-2489 (2005).

22. Heesen, M., Bloemeke, B., Heussen, N., & Kunz D. Can the interleukin-6 response to endotoxin be predicted? Studies of the influence of a promoter polymorphism of the interleukin-6 gene, gender, the density of the endotoxin receptor CD14, and inflammatory cytokines. *Crit Care Med.* **30,** 664–669 (2002).

23. Hernandez-Guerrero, C. et al. In-vitro secretion of proinflammatory cytokines by human amniochorion carrying hyper-responsive gene polymorphisms of tumour necrosis factor-alpha and interleukin-1beta. *Mol Hum Reprod.* **9,** 625–629 (2003).

24. Huebinger, R. M. et al. IL-10 polymorphism associated with decreased risk for mortality after burn injury. *J Surg Res. 164,* e141–e145 (2010).

25. Huizinga, T. W. et al. Are differences in interleukin 10 production associated with joint damage? *Rheumatology (Oxford).* **39,** 1180–1188 (2000).

26. Hull, J., Thomson, A. & Kwiatkowski, D. Association of respiratory syncytial virus bronchiolitis with the interleukin 8 gene region in UK families. *Thorax.* **5,** 1023–1027 (2000).

27. Iacoviello, L. et al. Polymorphisms of the interleukin-1beta gene affect the risk of myocardial infarction and ischemic stroke at young age and the response of mononuclear cells to stimulation in vitro. *Arterioscler Thromb Vasc Biol.* **25,** 222–227 (2005).

28. Ioannidis, J. P. et al. Assessment of cumulative evidence on genetic associations: interim guidelines. *Int J Epidemiol.* **37,** 120–132 (2008).

29. Kilpinen, S., Hulkkonen, J., Wang, X. Y. & Hurme, M. The promoter polymorphism of the interleukin-6 gene regulates interleukin-6 production in neonates but not in adults. *Eur Cytokine Netw*. **12,** 62–68 (2001).

30. Kim, J. G. et al. Cytokine production by whole blood cells: relationship to interleukin gene polymorphism and bone mass. *J Korean Med Sci.* **20,** 1017–1022 (2005).

31. Kiszel, P., Makó, V., Prohászka, Z. and Cervenak, L. Interleukin-6 -174 promoter polymorphism does not influence IL-6 production after LPS and IL-1 beta stimulation in human umbilical cord vein endothelial cells. *Cytokine.* **40,** 17–22 (2007).

32. Knaus, W. A, Draper, E. A., Wagner, D. P. & Zimmerman, J. E. APACHE II: a severity of disease classification system. *Crit Care Med.* **13,** 818–829 (1985).

33. Koss, K., Satsangi, J., Fanning, G. C., Welsh, K. I. & Jewell, D. P. Cytokine (TNF alpha, LT alpha and IL-10) polymorphisms in inflammatory bowel diseases and normal controls: differential effects on production and allele frequencies. *Genes Immun.* **1,** 185–190 (2000).

34. Kumpf, O. et al. Influence of genetic variations in TLR4 and TIRAP/Mal on the course of sepsis and pneumonia and cytokine release: an observational study in three cohorts. *Crit. Care.* **14,** R103 (2010).

35. Larsen, M. H. et al. Genome-Wide Association Study of Genetic Variants in LPS-Stimulated IL-6, IL-8, IL-10, IL-1ra and TNF-α Cytokine Response in a Danish Cohort. *PLoS One.* **8,** e66262 (2013).

36. Latella, M. C., et al. Interleukin 1 gene cluster, myocardial infarction at young age and inflammatory response of human mononuclear cells. *Immunol Invest.* **38,** 203–219 (2009).

37. Martinez-Ocaña, J. et al. Plasma cytokine levels and cytokine gene polymorphisms in Mexican patients during the influenza pandemicA(H1N1)pdm09. *J Clin Virol.* **58,** 108–113 (2013).

38. Martin-Loeches, I. et al. Variants at the promoter of the interleukin-6 gene are associated with severity and outcome of pneumococcal community-acquired pneumonia. *Intensive Care Med.* **38,** 256–262 (2012).

39. Misch, E. A., Verbon, A., Prins, J. M., Skerrett, S. J. & Hawn, T. R. A TLR6 polymorphism is associated with increased risk of Legionnaires' disease. *Genes Immun.* **14,** 420–426 (2013).

40. Moens, L. et al. Toll-like receptor 2 and Toll-like receptor 4 polymorphisms in invasive pneumococcal disease. *Microbes Infect.* **9,** 15–20 (2007).

41. Moher, D., Liberati, A., Tetzlaff, J., Altman, D. G. & PRISMA Group. Preferred reporting items for systematic reviews and meta-analyses: the PRISMA statement. *PLoS Med.* **6,** e1000097 (2009).

42. Mörmann, M. et al. Mosaics of gene variations in the Interleukin-10 gene promoter affect interleukin-10 production depending on the stimulation used. *Genes Immun.* **5,** 246–255 (2004).

43. Moscovis, S. M. et al. Interleukin-10 and sudden infant death syndrome. *FEMS Immunol Med Microbiol.* **42,** 130–138 (2004).

44. Noss, E. H., Nguyen, H. N., Chang, S. K., Watts, G. F., & Brenner, M. B. Genetic polymorphism directs IL-6 expression in fibroblasts but not selected other cell types. *Proc Natl Acad Sci U S A.* **112,** 14948–14953 (2015).

45. Nur, B. G. et al. Single nucleotide polymorphism and production of IL-1β and IL-10 cytokines in febrile seizures. *Neuropediatrics*. **43,** 194–200 (2012).

46. Patel, J. A. et al. Interleukin-6−174 and tumor necrosis factor α−308 polymorphisms enhance cytokine production by human macrophages exposed to respiratory viruses. *J Interferon Cytokine Res.* **30,** 917–921 (2010).

47. Pereira, A. C. et al. Genetic, epidemiological and biological analysis of interleukin-10 promoter single-nucleotide polymorphisms suggests a definitive role for -819C/T in leprosy susceptibility. *Genes Immun.* 10, 174–180 (2009).

48. Pociot, F., Mølvig, J., Wogensen, L., Worsaae, H. & Nerup, J. A TaqI polymorphism in the human interleukin-1 beta (IL-1 beta) gene correlates with IL-1 beta secretion in vitro. *Eur J Clin Invest.* **22,** 396–402 (1992).

49. Remmers, E. F. et al. Genome-wide association study identifies variants in the MHC class I, IL10, and IL23R-IL12RB2 regions associated with Behçet's disease. *Nat Genet.* **42,** 698–702 (2010).

50. Reuss, E. et al. Differential regulation of interleukin-10 production by genetic and environmental factors--a twin study. *Genes Immun.* **3,** 407–413 (2002).

51. Rivera-Chavez, F. A., Peters-Hybki, D. L., Barber, R. C. & O´ Keefe, G. E. Interleukin-6 promotor haplotypes and interleukin-6 cytokine response. *Shock.* **20,** 218–223 (2003).

52. Romanova, E. N. & Govorin, A. V. TNF-alpha, IL-10, and eNOS gene polymorphisms in patients with influenza A/H1N1 complicated by pneumonia. *Ter Arkh.* **85,** 58–62 (2013).

53. Rosado, S. et al. Interleukin-10 promoter polymorphisms in patients with systemic lupus erythematosus from the Canary Islands. *Int J Immunogenet.* **35,** 235–242 (2008).

54. Sakamoto, K. et al. Relationship between cytokine gene polymorphisms and risk of postoperative pneumonia with esophageal cancer. *J Gastrointest Surg.* **18,** 1247–1253 (2014).

55. Salhi, A. et al. Immunological and genetic evidence for a crucial role of IL-10 in cutaneous lesions in humansinfected with Leishmania braziliensis. *J Immunol.* **180,** 6139–6148 (2008).

56. Salnikova, L. E., Smelaya, T. V., Moroz, V. V., Golubev, A. M. & Rubanovich, A. V. Functional polymorphisms in the CYP1A1, ACE, and IL-6 genes contribute to susceptibility to community-acquired and nosocomial pneumonia. *Int J Infect Dis.* **17,** e433–e442 (2013).

57. Schaaf, B. et al. The interleukin-6 -174 promoter polymorphism is associated with extrapulmonary bacterial dissemination in Streptococcus pneumoniae infection. *Cytokine.* **31,** 324–328 (2005).

58. Schaaf, B. M. et al. Pneumococcal septic shock is associated with the interleukin-10-1082 gene promoter polymorphism. *Am J Respir Crit Care Med.* **168,** 476–480 (2003).

59. Schippers, E. F. et al. IL-10 and toll-like receptor-4 polymorphisms and the in vivo and ex vivo response to endotoxin. *Cytokine.* **29,** 215–228 (2005).

60. Schnetzke, U. et al. Polymorphisms of Toll-like receptors (TLR2 and TLR4) are associated with the risk of infectious complications in acute myeloid leukemia. *Genes Immun.* **16,** 83–88 (2015).

61. Schrijver, H. M., van As, J., Crusius, J. B., Dijkstra, C. D. & Uitdehaag, B. M. Interleukin (IL)-1 gene polymorphisms: relevance of disease severity associated alleles with IL-1beta and IL-1raproduction in multiple sclerosis. *Mediators Inflamm.* **12,** 89–94 (2003).

62. Stanilova, S. A., Miteva, L. D., Karakolev, Z. T. & Stefanov, C. S. Interleukin-10-1082 promoter polymorphism in association with cytokine production and sepsis susceptibility. *Intensive Care Med*. 32, 260–266 (2006).

63. Stappers, M. H. et al. Polymorphisms in cytokine genes IL6, TNF, IL10, IL17A and IFNG influence susceptibility to complicated skin and skin structure infections. *Eur J Clin Microbiol Infect Dis.* **33,** 2267–2274 (2014).

64. Steinke, J. W., Barekzi, E., Huyett, P. & Borish, L. Differential interleukin-10 production stratified by -571 promoter polymorphism in purified human immunecells. *Cell Immunol.* **249,** 101–107 (2007).

65. Stroup, D. F. et al. Meta-analysis of observational studies in epidemiology: a proposal for reporting. Meta-analysis Of Observational Studies in Epidemiology (MOOSE) group. *JAMA.* **283**, 2008–2012 (2000).

66. Suárez, A., Castro, P., Alonso, R., Mozo, L. & Gutiérrez, C. Interindividual variations in constitutive interleukin-10 messenger RNA and protein levels and their association with genetic polymorphisms. *Transplantation.* **75,** 711–717 (2003).

67. Tellería-Orriols J.J., García-Salido A., Varillas D., Serrano-González A., Casado-Flores J. TLR2-TLR4/CD14 polymorphisms and predisposition to severe invasive infections by Neisseria meningitidis and Streptococcus pneumoniae. Med. Intensiva. 2014;38(6):356–62. doi:10.1016/j.medin.2013.08.006.

68. Temple, S. E. et al. Alleles carried at positions -819 and -592 of the IL10 promoter affect transcription following stimulation of peripheral blood cells with Streptococcus pneumoniae. *Immunogenetics.* **55,** 629–632 (2003).

69. Vamvakopoulos, J., Green, C. & Metcalfe, S. Genetic control of IL-1beta bioactivity through differential regulation of the IL-1 receptor antagonist. *Eur J Immunol.* **32,** 2988–2996 (2002).

70. Vincent, J. L. et al. The SOFA (sepsis-related organ failure assessment) score to describe organ dysfunction/failure. *Intensive Care Med.* **22,** 707–710 (1996).

71. Wan, Q. Q., Li, J. L., Ye, Q. F., Zhou, J. D. Genetic association of tumor necrosis factor-beta, interleukin-10, and interleukin-1 gene cluster polymorphism with susceptibility to pneumonia in kidney transplant recipients. *Transplant Proc.* **45,** 2211–2214 (2013).

72. Wattanathum, A., Manocha, S., Groshaus, H., Russell, J. A. & Walley, K. R. Interleukin-10 haplotype associated with increased mortality in critically ill patients with sepsis from pneumonia but not in patients with extrapulmonary sepsis. *Chest.* **128,** 1690–1698 (2005).

73. Wen, A. Q. et al. Effects of haplotypes in the interleukin 1beta promoter on lipopolysaccharide-induced interleukin 1beta expression. *Shock.* **26,** 25–30 (2006).

74. Yan, Z. et al. Regulatory polymorphisms in the IL-10 gene promoter and HBV-related acute liver failure in the Chinese population. *J Viral Hepat.* **16,** 775–783 (2009).

75. Yilmaz, V., Yentür, S. P. & Saruhan-Direskeneli, G. IL-12 and IL-10 polymorphisms and their effects on cytokine production. *Cytokine.* **30,** 188–194 (2005).

76. Yuan, F. F. et al. Clinical relevance of TLR2, TLR4, CD14 and FcgammaRIIA gene polymorphisms in Streptococcus pneumonia infection. *Immunol Cell Biol.* **86,** 268–270 (2008).

77. Zeng, L. et al. Clinical relevance of the interleukin 10 promoter polymorphisms in Chinese Han patients with major trauma: genetic association studies. *Crit Care.* **13,** R188 (2009).

78. Zidan, H. E., Elbehedy, R. M. & Azab, S. F. Il6-174 g/c gene polymorphism and its relation to serum il6 in egyptian children with community-acquired pneumonia. *Cytokine.* **67,** 60–64 (2014).
